# Dual Regulation of Gene Expression Mediated by Extended MAPK Activation and Salicylic Acid Contributes to Robust Innate Immunity in *Arabidopsis thaliana*

**DOI:** 10.1371/journal.pgen.1004015

**Published:** 2013-12-12

**Authors:** Kenichi Tsuda, Akira Mine, Gerit Bethke, Daisuke Igarashi, Christopher J. Botanga, Yayoi Tsuda, Jane Glazebrook, Masanao Sato, Fumiaki Katagiri

**Affiliations:** 1 Department of Plant Microbe Interactions, Max Planck Institute for Plant Breeding Research, Cologne, Germany; 2 Department of Plant Biology, Microbial and Plant Genomics Institute, University of Minnesota, St. Paul, Minnesota, United States of America; 3 Institute for Innovation, Ajinomoto Co., Inc., 1-1, Suzuki-cho, Kawasaki-ku, Kawasaki, Japan; 4 Department of Biological Sciences, Chicago State University, Chicago, Illinois, United States of America; 5 National Institute for Basic Biology, Okazaki Institute for Integrative Bioscience, National Institutes of Natural Sciences, Higashiyama, Myodaiji, Okazaki, Japan; Unité de Recherche en Genomique Végétale, France

## Abstract

Network robustness is a crucial property of the plant immune signaling network because pathogens are under a strong selection pressure to perturb plant network components to dampen plant immune responses. Nevertheless, modulation of network robustness is an area of network biology that has rarely been explored. While two modes of plant immunity, Effector-Triggered Immunity (ETI) and Pattern-Triggered Immunity (PTI), extensively share signaling machinery, the network output is much more robust against perturbations during ETI than PTI, suggesting modulation of network robustness. Here, we report a molecular mechanism underlying the modulation of the network robustness in *Arabidopsis thaliana*. The salicylic acid (SA) signaling sector regulates a major portion of the plant immune response and is important in immunity against biotrophic and hemibiotrophic pathogens. In *Arabidopsis*, SA signaling was required for the proper regulation of the vast majority of SA-responsive genes during PTI. However, during ETI, regulation of most SA-responsive genes, including the canonical SA marker gene *PR1*, could be controlled by SA-independent mechanisms as well as by SA. The activation of the two immune-related MAPKs, MPK3 and MPK6, persisted for several hours during ETI but less than one hour during PTI. Sustained MAPK activation was sufficient to confer SA-independent regulation of most SA-responsive genes. Furthermore, the MPK3 and SA signaling sectors were compensatory to each other for inhibition of bacterial growth as well as for *PR1* expression during ETI. These results indicate that the duration of the MAPK activation is a critical determinant for modulation of robustness of the immune signaling network. Our findings with the plant immune signaling network imply that the robustness level of a biological network can be modulated by the activities of network components.

## Author Summary

Robustness of a network is defined by how consistently it performs upon removal of some of its components. It is a common strategy for plant pathogens to attack components of the plant immune signaling network in an attempt to dampen plant immunity. Therefore, it is crucial for the plant immune signaling network to have a high level of robustness. We previously reported that the robustness level of the plant immune signaling network is higher during Effector-Triggered Immunity (ETI) than Pattern-Triggered Immunity (PTI). Here we discovered a molecular switch that determines two robustness levels during ETI and PTI. Salicylic acid (SA) is a major plant immune signal molecule that regulates many immune-related genes. SA-independent alternative mechanisms also regulated the majority of SA-responsive genes during ETI but not PTI. One of the SA-independent mechanisms was mediated by prolonged activation of MAP kinases (MAPKs). MAPK activation was prolonged during ETI but transient during PTI. Thus, the duration of MAPK activation switches the robustness level of the plant immune signaling network. Our findings imply that the robustness level of a biological network can be modulated by activities of its components.

## Introduction

How network properties, such as robustness against network perturbations, emerge from biological networks has been a central question in systems biology [1], [2]. Possible modulation of network robustness in a biologically relevant context and mechanisms underlying the modulation are areas of study that have rarely been explored.

Innate immunity, in which defense responses are induced through signaling events initiated by recognition of pathogen attack, composes a major part of plant immunity [3]. PAMP/Pattern-Triggered Immunity (PTI) and Effector-Triggered Immunity (ETI) are modes of plant innate immunity defined by the way pathogens are detected [4], [5]. PTI is triggered by recognition of microbe/pathogen-associated molecular patterns (MAMPs/PAMPs) by the cognate pattern-recognition receptors (PRRs), which are typically receptor-like kinases or receptor-like proteins [6]. For example, *Arabidopsis thaliana* FLS2 is the PRR for flg22, an elicitor-active epitope of flagellin from Gram-negative bacteria [7]. While most non-adapted pathogens cannot overcome PTI, adapted pathogens deliver effectors into the plant cell that manipulate plant cell functions to facilitate their infection by, for instance, interfering with PTI signaling [8], [9]. ETI is triggered by specific recognition of effectors by resistance (R) proteins, which are often nucleotide-binding leucine-rich repeat (NB-LRR) proteins [10]. For example, the *Arabidopsis* intracellular NB-LRR R proteins RPS2 and RPM1 indirectly recognize perturbations of the PTI signaling component RIN4 by the effectors AvrRpt2 and AvrRpm1/AvrB, respectively, of a Gram-negative bacterial pathogen, *Pseudomonas syringae* [3]. In addition to proteinaceous effectors, some *P. syringae* strains deliver coronatine, which is a jasmonic isoleucine mimic, in order to suppress plant immunity [11]. Recently, it was shown that coronatine suppresses immune responses dependent on salicylic acid (SA) as well as independent of SA [12], [13]. Thus, there are evolutionary arms races between hosts and pathogens. Pathogens evolve much faster than hosts, rapidly changing effector repertoires, thereby changing points of attack in host immune networks. As hosts cannot match the speed of pathogen evolution, it is important that hosts develop robust immune networks that remain functional in the face of effector attack. Mechanisms underlying network robustness are thus a critical aspect of immunity.

SA is a signal molecule controlling a major portion of immunity against biotrophic and hemibiotrophic pathogens, including *P. syringae* [14]. *SID2* encodes a key enzyme for SA biosynthesis in response to pathogen infection [15]. In *Arabidopsis sid2* mutants, pathogen-induced SA accumulation is almost undetectable [14]. Hundreds of genes are transcriptionally regulated by SA signaling, mediated mainly by a positive regulator of SA signaling, NPR1 [14]. *PR1* is one SA-inducible gene used as a canonical SA marker [14].

*Arabidopsis* has 20 mitogen-activated protein kinases (MAPKs) [16], and four of them, MPK3, MPK4, MPK6 and MPK11, have been described as immune signaling components [17]. MPK3 and MPK6 are associated with immune responses, such as reactive oxygen species (ROS) production, ET production/signaling, phytoalexin production and cell death [17]. For instance, ethylene production is positively controlled by dual regulation of enzymes (ACS) synthesizing the ethylene precursor 1-amino-cyclopropane-1-carboxylic acid. MPK6 stabilizes ACS2 and ACS6 by their phosphorylation, and MPK3 and MPK6 control gene expression through a transcription factor, WRKY33, which is activated by the MAPKs [18], [19]. The same cascade is required for production of a phytoalexin, camalexin, by controlling expression of a biosynthetic gene, *PAD3* [20]. A double mutant deficient in *MPK3* and *MPK6* is embryonic lethal but the single mutants are viable, suggesting functional redundancy between them in development [21]. MPK3 phosphorylates the bZIP type transcription factor VIP1 whose phosphorylation is required for its nuclear translocation [22]. Transient over-expression of VIP1 led to weak induction of *PR1* in *Arabidopsis* protoplasts although involvement of SA in this *PR1* induction is not known [23].

The overall spectra of induced defense responses are overlapping between PTI and ETI whereas the kinetics and intensity of the responses seem different [4], [24]. In *Arabidopsis*, knocking out the hub genes of four major signaling sectors abolished 80% of flg22-triggered PTI (flg22-PTI) and AvrRpt2-triggered ETI (AvrRpt2-ETI), indicating extensively shared signaling network machinery between PTI and ETI [25]. Relationships among these signaling sectors are part compensatory and part synergistic in flg22-PTI but are predominantly compensatory in AvrRpt2-ETI, which explains a high level of robustness in the ETI level against network perturbations [25]. Single mutations (*dde2*, *ein2*, *pad4* and *sid2*) weakly but significantly compromised flg22-PTI but not AvrRpt2-ETI while the quadruple mutation largely abolished both. These observations demonstrated differences in the robustness of the highly overlapping signaling networks during the two modes of plant immunity. However, the molecular mechanism controlling modulation of the network robustness is not known.

Here we report a molecular mechanism that affects the robustness of the plant immune signaling network. Although *Arabidopsis* MPK3 and MPK6 are activated during both PTI and ETI, the duration of the activation was much longer during ETI than PTI. Only sustained activation of the MAPKs supported expression of a majority of SA-responsive genes in the absence of SA. The roles of MPK3 and SA signaling during AvrRpt2-ETI were compensatory, contributing to network robustness against perturbations during ETI. Our findings demonstrate that a biologically important differential network property, robustness, can emerge from duration of the activity of a network component.

## Results

### Most SA-responsive genes were properly regulated in the absence of SA during ETI

**Figure 1:**
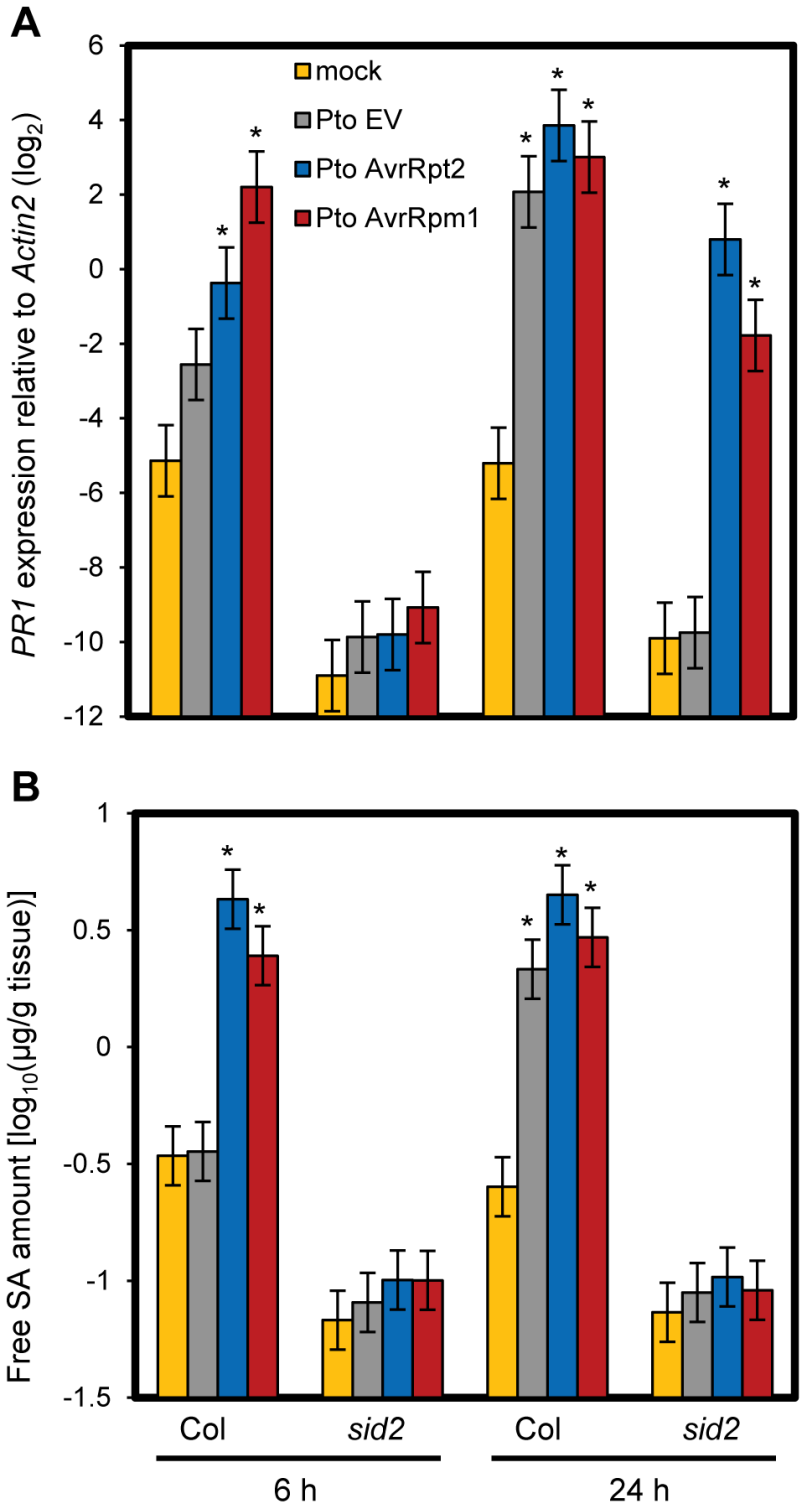
SA-independent regulation of *PR1* during ETI. (A) The *PR1* expression level in leaves at 6 or 24 hpi with *Pto* strains (OD_600_ = 0.001) or mock was determined by qRT-PCR. Bars represent means and standard errors of two biological replicates calculated using a mixed linear model. The vertical axis shows the log_2_ expression level relative to *Actin2* (At2g18780). (B) The free SA levels in leaf samples corresponding to those in (A) were determined. Bars represent means and standard errors of two biological replicates calculated using a mixed linear model. The SA level is shown on a log_10_ scale. Asterisks indicate significant differences from mock (*P*<0.01, two-tailed *t*-tests).

We previously reported that ETI is more robust against network perturbations than PTI due to a higher level of network compensation [25]. We hypothesized that this compensation occurred at the level of gene regulation. To test this hypothesis, we examined expression of a canonical SA marker gene, *PR1*, during ETI. Transcriptional induction of *PR1* was completely dependent on SID2, which is a key SA biosynthetic enzyme, and hence completely dependent on SA signaling during PTI [26]. We found that *PR1* induction was only partially dependent on SID2 and NPR1 at a late time point of 24 hours post inoculation (hpi) with ETI-triggering *P. syringae* pv. *tomato* DC3000 (*Pto*) strains expressing the effectors AvrRpt2 (*Pto* AvrRpt2) or AvrRpm1 (*Pto* AvrRpm1) (Figure 1A and Figure S1). While AvrRpt2 and AvrRpm1 are recognized by the CC-type NB-LRR proteins RPS2 and RPM1, AvrRps4 is recognized by the TIR-type NB-LRR protein RPS4 [3]. We also observed SID2- and NPR1-independent *PR1* induction during AvrRps4-triggered ETI although induction levels were lower compared to AvrRpt2- and AvrRpm1-ETI (Figure S1). In contrast, *PR1* induction was completely dependent on SID2 in the case of the non-ETI triggering *Pto* strain carrying an empty vector (*Pto* EV). Inoculation of the ETI-triggering strains at a high dose can trigger a form of programmed cell death called a hypersensitive response (HR) [3]. The inoculation dose used in this experiment was relatively low (OD_600_ = 0.001), and we did not observe a macroscopic HR within 24 hpi. To test the possibility that the SA level increased independently of SID2 during ETI, we measured the SA level in these tissues. The increased SA accumulation was completely dependent on SID2 in all conditions (Figure 1B). These results indicate that some SA-independent mechanism(s) can activate *PR1* during ETI. At an earlier time point of 6 hpi, only SA-dependent *PR1* induction was observed with all three strains (Figure 1A), suggesting that this SA-independent mechanism(s) during ETI requires more than 6 hours to be effective.

**Figure 2:**
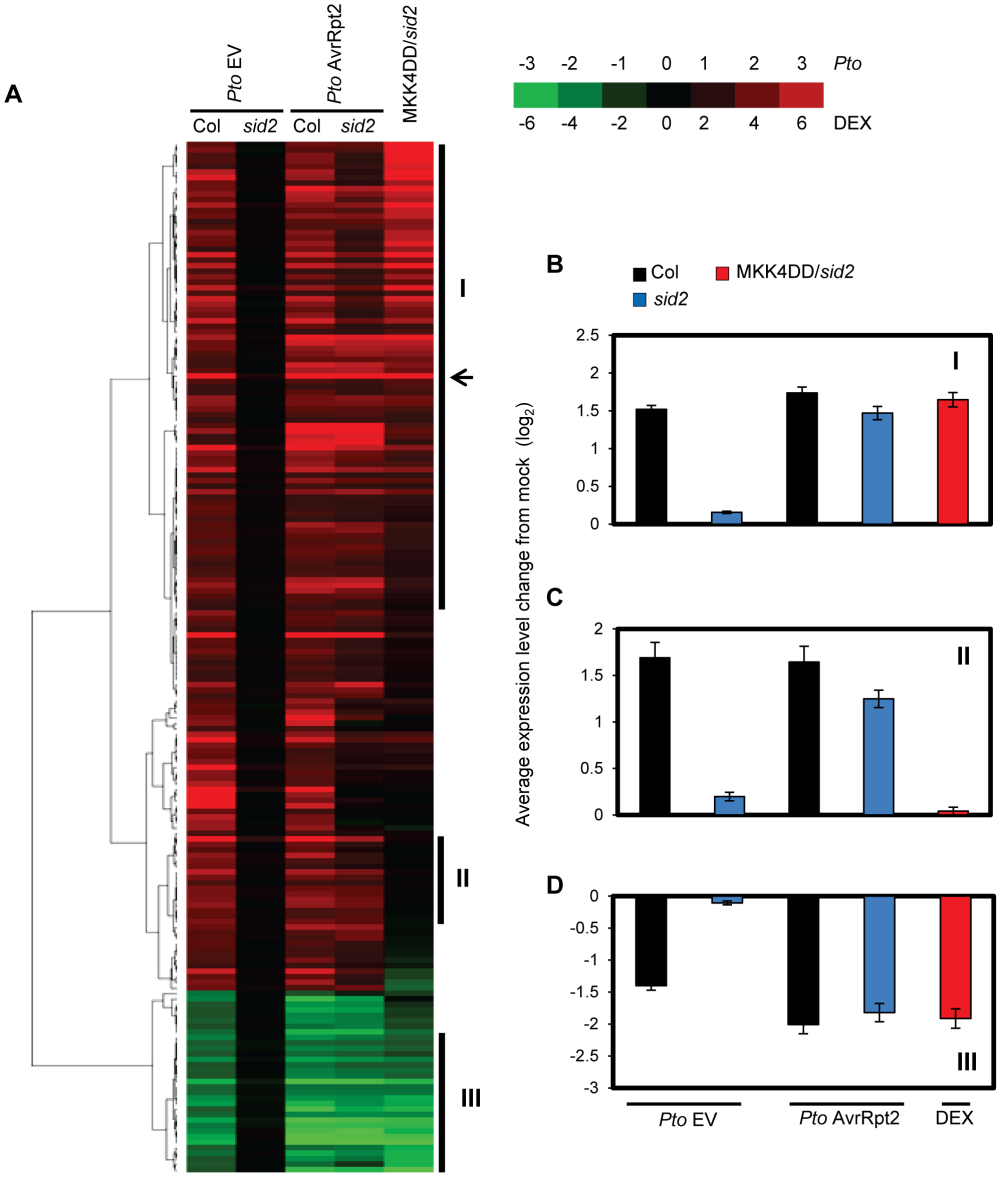
Sustained MAPK activation supports transcriptional regulation of a majority of SA-responsive genes without SA. (A). A heatmap of the SA-responsive genes. Leaves were collected at 24 hpi with the indicated *Pto* strains (OD_600_ = 0.001) or mock. Independently, leaves of *DEX-MKK4DD* plants were collected at 24 hpi with 2 µM DEX or mock and subjected to mRNA profiling analysis using a whole genome DNA microarray. SA-responsive genes were selected for reproducible SID2-dependent responsiveness to the *Pto* strains as described in Experimental Procedures. The log_2_ ratios compared to mock for 187 SA-responsive genes were subjected to agglomerative hierarchical clustering analysis. The log_2_ ratio of DEX/mock for the *DEX-MKK4DD sid2* plant (MKK4DD/*sid2*) samples was weighted by a factor of 0.5 to reduce its effects on the clustering pattern. The log_2_ ratios used were averaged from three independent experiments. Green indicates negative values, red indicates positive values and black indicates zero: see the color scale. The arrow indicates the position of *PR1*. Means and standard errors of (B) Cluster I, 85 genes; (C) Cluster II, 20 genes; (D) Cluster III, 25 genes are shown.

SA-independent mechanism(s) for *PR1* induction during ETI prompted us to investigate the possibility that other SA-responsive genes can also be transcriptionally regulated in an SA-independent manner during ETI. For this purpose, mRNA profiles were analyzed using a whole genome DNA microarray. Leaves of wild type (Col) or *sid2* plants were inoculated with water (mock), *Pto hrcC*, *Pto* EV, or *Pto* AvrRpt2, and were collected at 24 hpi for mRNA profiling. The *Pto hrcC* strain is deficient in the type III secretion system used to transport effectors into plant cells. It elicits the PTI response by presenting various MAMPs [11]. Among 2828 genes that were significantly up- or down-regulated (with *q* values<0.01 and more than 2-fold changes) in both *Pto* EV and *Pto* AvrRpt2 infection in Col, regulation of 187 genes showed strong SID2-dependence in *Pto* EV infection (Figure 2A and Table S1). These genes are designated SA-responsive genes hereafter. Remarkably, regulation of most SA-responsive genes, including *PR1*, at 24 hpi with *Pto* AvrRpt2 is largely SID2-independent although SA contributes to their full expression, indicating that SA-independent signaling mechanism(s) can regulate most SA-responsive genes during AvrRpt2-ETI. The SID2-dependency of gene regulation after *Pto hrcC* inoculation was similar to that after *Pto* EV inoculation, although the overall extent of up- or down-regulation was lower, and distinct from that after *Pto* AvrRpt2 inoculation (Figure S2 and Table S2). Thus, initiation of ETI appears to be the key for activation of this SA-independent mechanism(s).

### Activation of MPK3 and MPK6 was sustained in ETI but transient in non-ETI

**Figure 3:**
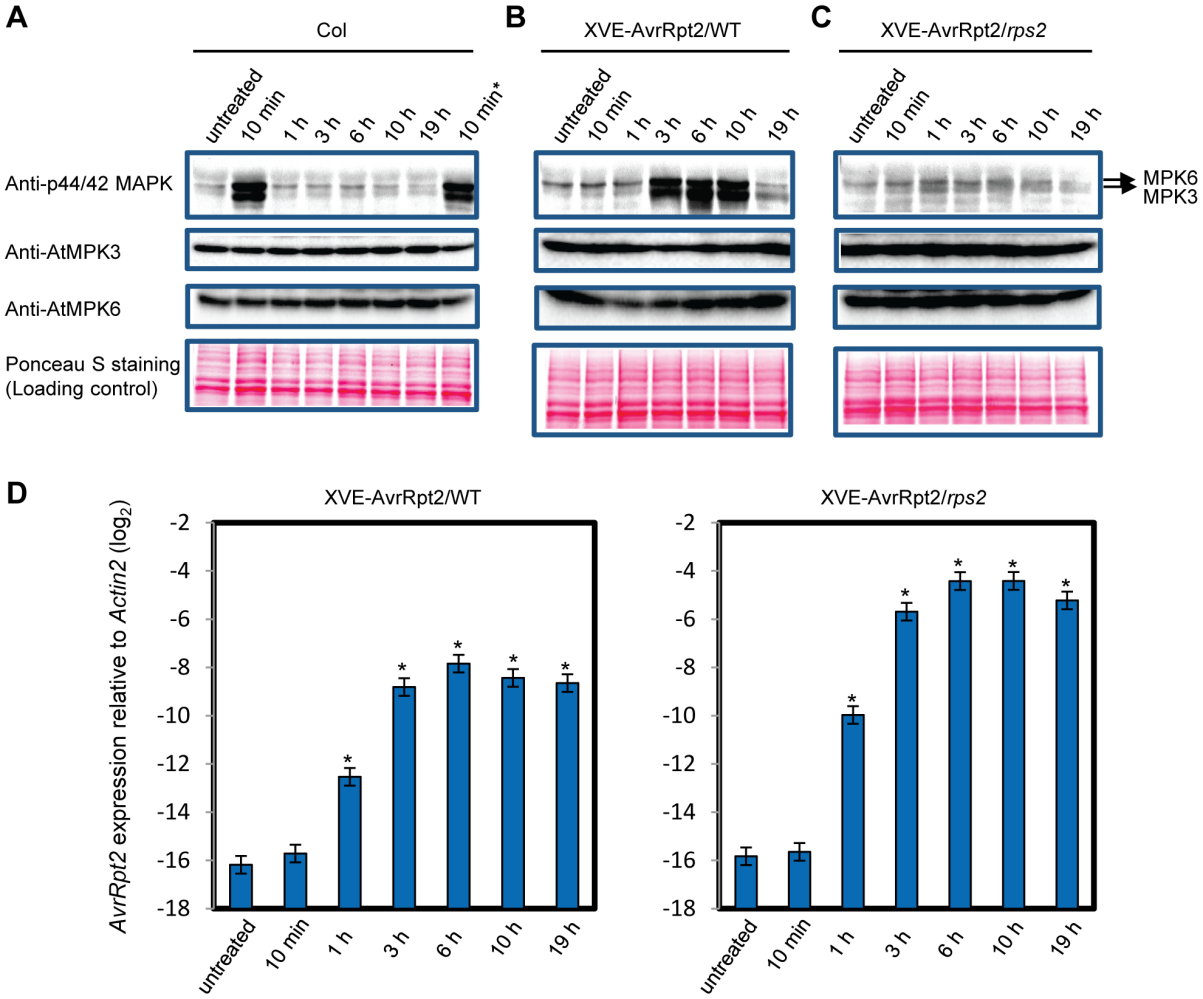
MAPK activation is sustained in ETI but transient in PTI. (A) MAPK activation during flg22-PTI. Seedlings were treated with 10 nM flg22 for the indicated times in a liquid medium. For the 10 min* sample, fresh seedlings were treated with the flg22-containing liquid medium used for the 3 h sample, revealing that flg22 was not degraded in the 3 h sample. (B,C) MAPK activation during AvrRpt2-ETI. Seedlings of the transgenic lines that carry the estradiol-inducible *AvrRpt2* transgene in wild-type (B) or *rps2* (C) background (*XVE-AvrRpt2*/WT or *rps2*) were treated with 20 nM estradiol for the indicated times in a liquid medium. Activated MAPKs, MPK3 and MPK6 were detected by immunoblot using anti-p44/42 MAPK, anti-AtMPK3 and anti-AtMPK6 antibody, respectively. Ponceau S stained blots are shown for loading controls. Experiments were conducted three times with similar results. (D) The *AvrRpt2* mRNA levels in seedlings treated with 20 nM estradiol for the indicated times were determined by qRT-PCR. Bars represent means and standard errors of two biological replicates calculated using a mixed linear model. The vertical axis shows the log_2_ expression level relative to *Actin2* (At2g18780). Asterisks indicate significant differences from the untreated controls (*P*<0.01, two-tailed *t*-tests).

**Figure 4:**
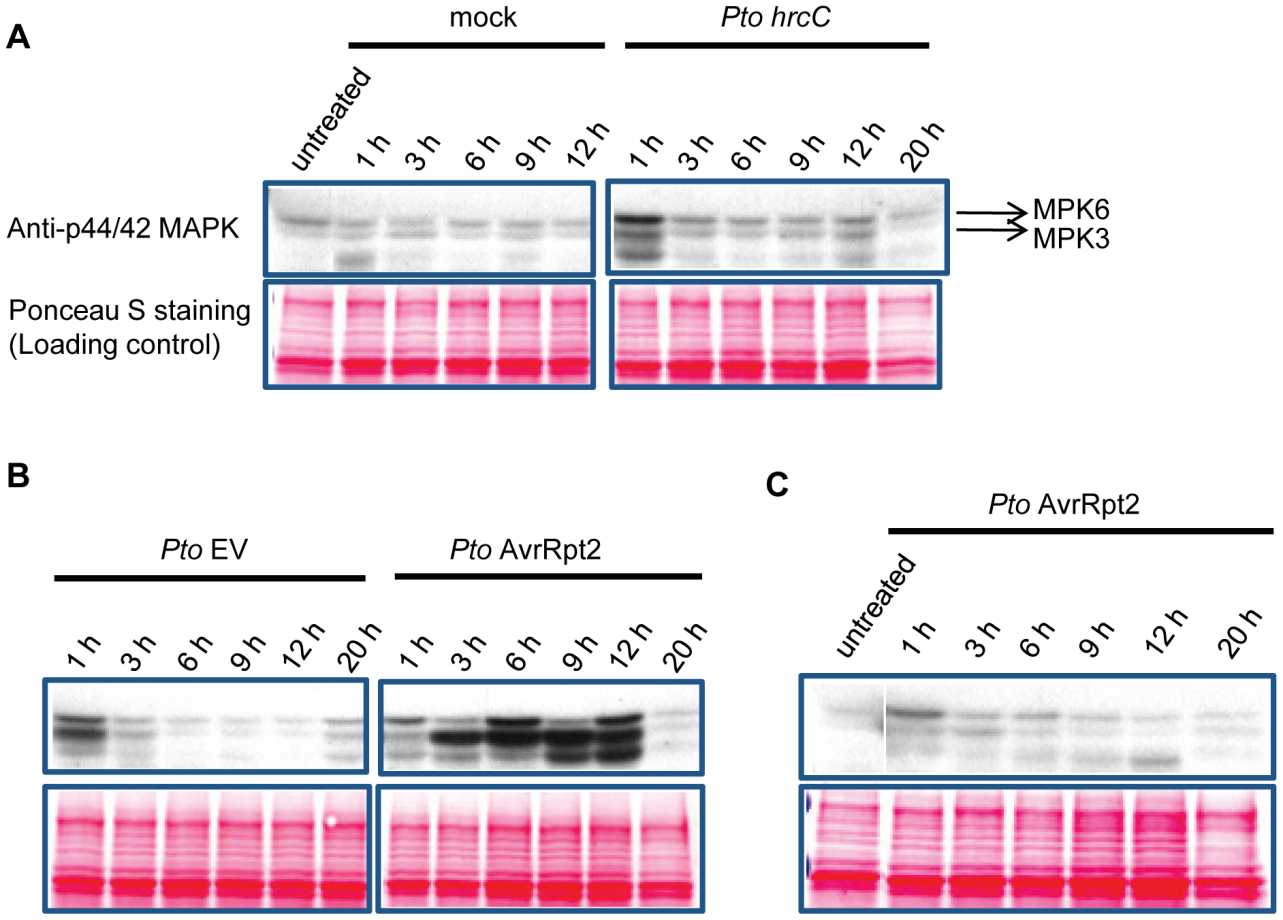
MAPK activation is sustained in ETI but transient in non-ETI conditions. Leaves of Col (A, B) and *rpm1 rps2* (C) plants were infiltrated with *Pto hrcC*, *Pto* EV, *Pto* AvrRpt2 (OD_600_ = 0.01) or water (mock) and samples were collected at the indicated time points. Activated MAPKs were detected by immunoblot using anti-p44/42 MAPK antibody. Ponceau S stained blots are shown for loading controls. Experiments were conducted three times, yielding similar results.

We hypothesized that a kinetic difference in activation of network components is responsible for activation of SA-independent mechanism(s). A prior study suggested that the duration of MPK3 and MPK6 activation is longer during ETI than non-ETI [27]. We compared the duration of MAPK activation in ETI and PTI. When wild-type seedlings in a liquid medium were treated with the PTI inducer flg22, activation of the MAPKs was observed after 10 min and returned to the basal level within one hour (Figure 3A), confirming previous observations [28]. The possibility that flg22 was rapidly degraded in the liquid culture was excluded since the MAPKs were activated similarly when fresh seedlings were placed in the liquid medium containing flg22 that had been incubated with other seedlings for 3 hours (Figure 3A). Thus, MAPK activation is truly transient after flg22 treatment. We employed transgenic seedlings carrying an estradiol-inducible *AvrRpt2* transgene (*XVE-AvrRpt2*) to measure MAPK activation during ETI in the absence of PTI. The MAPKs were activated by three hours and remained active for at least 7 hours after estradiol treatment (Figure 3B). This sustained MAPK activation was ETI-specific as no such activation was observed in the *rps2* mutant background, which lacks the corresponding receptor (Figure 3C). *PR1* induction during AvrRpt2-ETI was independent of SA in *XVE-AvrRpt2* transgenic seedlings (Figure S3), which is consistent with the results obtained using adult leaves inoculated with a *Pto* strain expressing AvrRpt2 (Figure 1). Similar trends in MAPK activation duration were observed when adult leaves were inoculated with *Pto* strains: sustained activation of the MAPKs was observed with *Pto* AvrRpt2 in a manner dependent on the *R* gene *RPS2*, but not with the strains that do not trigger ETI (Figure 4). While the amounts of activated MPK3 and MPK6 were similar during AvrRpt2-ETI triggered in *XVE-AvrRpt2* transgenic plants (Figure 3), there was more activated MPK3 than activated MPK6 during AvrRpt2-ETI triggered by *Pto* AvrRpt2 (Figures 4, S4 and S5), suggesting that MPK3 plays a major role during AvrRpt2-ETI in bacterial infection. We also observed sustained MAPK activation during AvrRps4-ETI although levels of activation were weaker compared to AvrRpt2-ETI (Figure S4). Since there are 20 MAPKs in *Arabidopsis* [16], we determined the identities of the activated MAPKs. Indeed, the activated MAPKs during AvrRpt2- and AvrRps4-ETI were MPK3 and MPK6 (Figure S4). Previously, Beckers et al (2009) reported that an SA analog, benzo(1,2,3,)thiadiazole-7-carbothioic acid S-methyl ester (BTH), induced priming of MPK3 activation by inducing expression of *MPK3* [29]. In contrast, sustained activation of MPK3 during AvrRpt2-ETI was independent of SA (Figure S5). The sustained activation was not due to an increased amount of MPK3 as we did not observe obvious changes in the MPK3 protein level during AvrRpt2-ETI (Figure 3). Taken together, our data show that sustained activation of the MAPKs is SA-independent and occurs during ETI but not during non-ETI responses.

### Sustained activation of MPK3 and MPK6 is sufficient for *PR1* induction in the absence of SA

**Figure 5:**
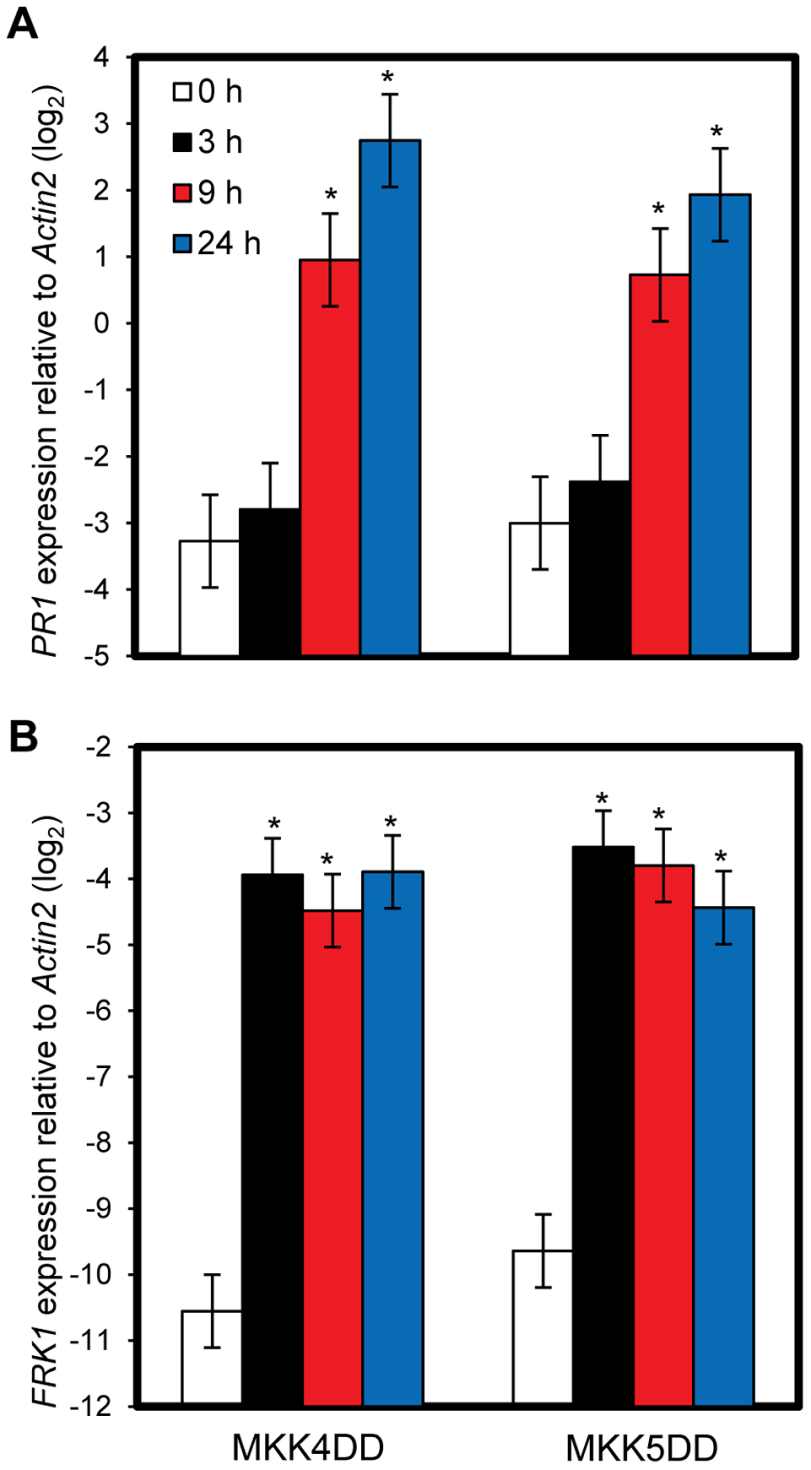
Sustained MAPK activation is sufficient for *PR1* induction. The *PR1* (A) or *FRK1* (B) expression levels in *DEX-MKK4DD* (MKK4DD) or *-MKK5DD* (MKK5DD) at the indicated times after treatment with 2 µM DEX were determined by qRT-PCR. Bars represent means and standard errors of three biological replicates calculated using a mixed linear model. The vertical axis shows the log_2_ expression level relative to *Actin2* (At2g18780). Asterisks indicate significant differences from untreated samples (0 h) (*P*<0.01, two-tailed *t*-tests).

**Figure 6:**
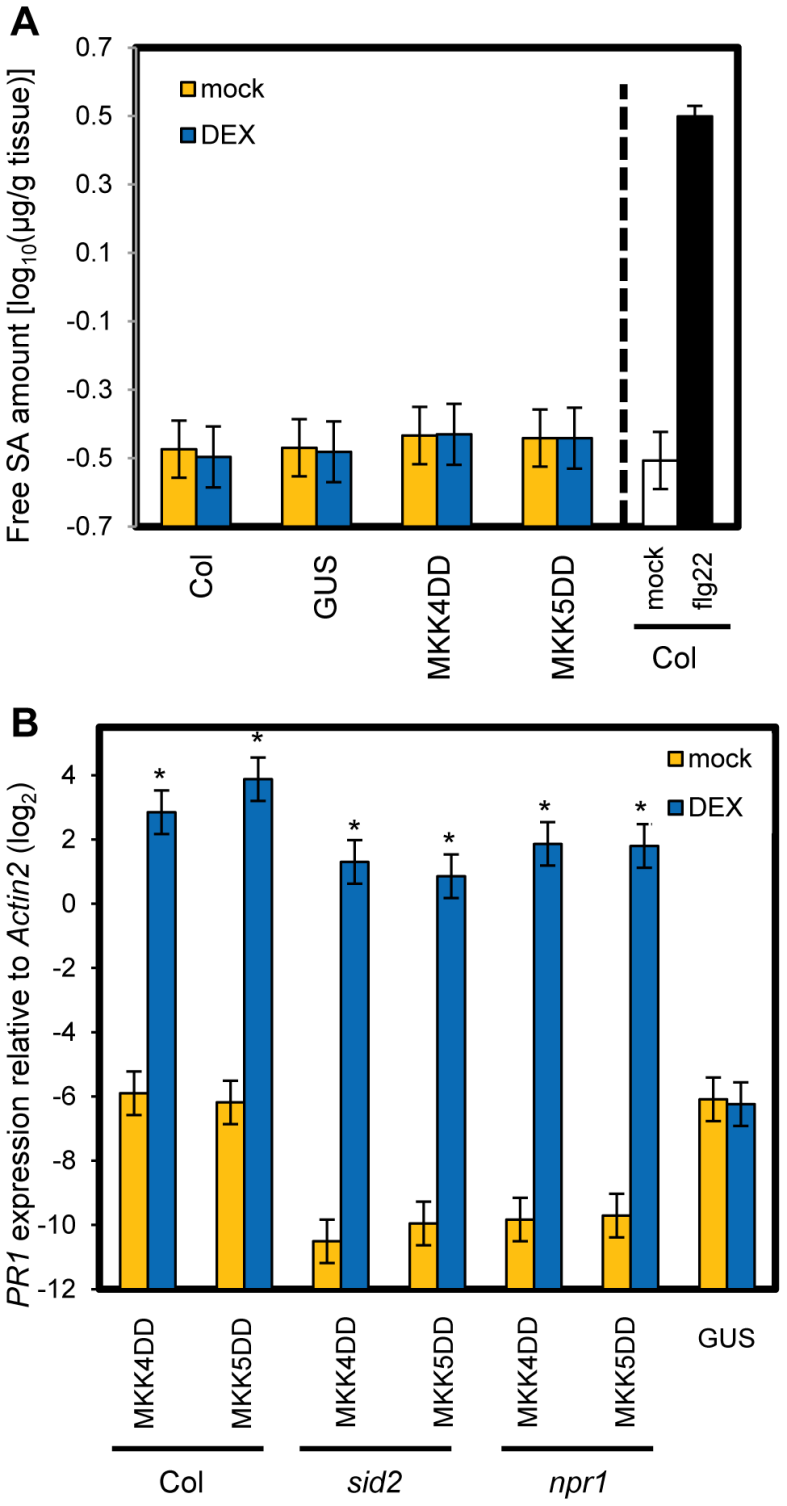
SA signaling is not involved in *PR1* induction by sustained MAPK activation. (A) The free SA levels in leaves of wild-type (Col) or *DEX-GUS* (GUS), *-MKK4DD* (MKK4DD) or *-MKK5DD* (MKK5DD) plants 9 hours after treatment with 2 µM DEX (DEX) or mock. For the Col (flg22) sample, leaves of Col plants were infiltrated with 1 µM flg22 or mock, and the result is shown as a positive control for induced SA accumulation. Bars represent means and standard errors of four biological replicates calculated using a mixed linear model. The SA level is shown on a log_10_ scale. (B) The *PR1* expression levels in leaves of the plant lines carrying the *DEX-GUS* (GUS), *-MKK4DD* (MKK4DD) or *-MKK5DD* (MKK5DD) transgenes in wild-type (Col), *sid2* or *npr1* backgrounds 24 hours after treatment with 2 µM DEX (DEX) or mock were determined by qRT-PCR. Bars represent means and standard errors of two biological replicates calculated using a mixed linear model. The vertical axis shows the log_2_ expression level relative to *Actin2* (At2g18780). Asterisks indicate significant differences from mock (*P*<0.01, two-tailed *t*-tests).

To test if sustained activation of MPK3 and MPK6 can induce *PR1* in an SA-independent manner, transgenic plants expressing constitutively active forms of MKK4 (MKK4DD) or MKK5 (MKK5DD) under the control of a dexamethasone (DEX)-inducible promoter were employed (*DEX-MKK4DD* and *DEX-MKK5DD*). MKK4 and MKK5 are MAP kinase kinases, whose activated forms phosphorylate and activate MPK3 and MPK6 [17]. DEX-induced expression of MKK4DD or MKK5DD leads to sustained activation of MPK3 and MPK6 (Figure S6) [30]. Induction of *PR1* was observed 9 hours after DEX treatment (Figure 5A), suggesting that sustained activation of MPK3 and MPK6 is sufficient for induction of *PR1*. Induction of *FRK1* is thought to be a good marker for activation of MPK3 and MPK6 [31] and was observed 3 hours after DEX treatment while *PR1* was not (Figure 5B). *FRK1* was strongly induced 30 minutes after flg22 treatment [32], and the induction did not require SA accumulation (Figure S7). Thus, although transient MAPK activation of MPK3 and MPK6 is sufficient for *FRK1* induction, sustained MAPK activation is necessary and sufficient for SA-independent *PR1* induction. The sustained activation of MPK3 and MPK6 by DEX-induced MKK4DD or MKK5DD did not increase the level of SA (Figure 6A). Furthermore, a wild-type-like *PR1* induction 24 hours after DEX treatment was observed in plants deficient in *SID2* or *NPR1* (Figure 6B). Since *PR1* induction was not observed in a DEX-inducible ß-glucuronidase (*GUS*, an arbitrary reporter gene) line after DEX treatment, *PR1* induction was not caused by the DEX-inducible system or DEX but by induced expression of MKK4DD or MKK5DD. Although MPK4 was activated as well as MPK3 and MPK6 during PTI and ETI (Figure 4; [17]), expression of MKK4DD or MKK5DD does not lead to strong activation of MPK4 [30]. Therefore, it is unlikely that MPK4 plays a role. We conclude that sustained activation of MPK3 and/or MPK6 causes *PR1* induction in an SA-independent manner.

We tested whether *mpk3* and *mpk6* single mutations had effects on *PR1* induction by MKK4DD or MKK5DD expression. *PR1* induction was unaffected in *mpk6* but strongly reduced in *mpk3* plants (Figure S8A). *MKK4DD* induction was also strongly reduced in *mpk3* plants (Figure S8B), so the reduction of *PR1* induction in *DEX-MKK4DD/mpk3* may be due to reduction of *MKK4DD* expression. *MKK5DD* induction in *DEX-MKK5DD/mpk3* was reduced compared to *DEX-MKK5DD/*Col yet 10 times higher than *MKK4DD* induction in *DEX-MKK4DD/mpk3* while *PR1* induction was similarly compromised in both plant lines. Thus, these results suggest that MPK3 is required for SA-independent *PR1* induction conferred by forced MKK5 activation while MPK6 is dispensable.

### Sustained activation of MPK3 and MPK6 supported transcriptional regulation of most SA-responsive genes

We tested whether sustained activation of MPK3 and/or MPK6 also regulates other SA-responsive genes. Leaves of the *DEX-MKK4DD* transgenic lines in wild type (Col) or *sid2* backgrounds were treated with DEX or mock control and were collected for mRNA profiling at 24 hours after treatment. The transcriptomic changes caused by DEX treatment were very similar between Col and *sid2* (Figure S9 and Table S3), indicating that gene regulation by sustained activation of the MAPKs is mostly independent of SA. Therefore, only the mRNA profile from the *DEX-MKK4DD sid2* line was included in the following analysis. The heatmap in Figure 2A shows that a majority of the SA-responsive genes responded in the DEX-treated *DEX-MKK4DD sid2* line similarly to *sid2* plants during AvrRpt2-ETI: most up-regulated or down-regulated SA-responsive genes in *sid2* during AvrRpt2-ETI were up-regulated or down-regulated, respectively, in the DEX-treated *DEX-MKK4DD sid2* line. This suggests that sustained activation of the MAPKs regulates a majority of SA-responsive genes in an SA-independent manner during AvrRpt2-ETI.

Three gene clusters were selected for further analysis (Clusters I–III in Figure 2A). The expression level changes of genes in each cluster were averaged and shown in Figure 2B–D. Clusters I and III include genes up- or down-regulated, respectively, in a SID2-independent manner during AvrRpt2-ETI and by sustained activation of the MAPKs. Thus, these genes appear to be regulated by sustained activation of the MAPKs during ETI. Cluster II includes genes that were up-regulated in a largely *SID2*-independent manner during ETI but not up-regulated by sustained activation of the MAPKs. Thus, up-regulation of the Cluster II genes during ETI is supported by a mechanism(s) other than the mechanism mediated by the MAPKs. When the GO terms associated with the clusters were examined, Cluster I, but none of the other clusters, was enriched with genes related to biological stresses (response to biotic stimulus, *P* = 2.8×10^−5^; response to other organism, *P* = 1.1×10^−4^; multi-organism process, *P* = 5.9×10^−4^). The results imply that genes induced by both SA and the MAPKs are important for biological stress responses. The regulatory trends for the clusters were confirmed by qRT-PCR analysis of one gene from each cluster (Figure S10).

### Compensatory relationships between the MAPKs and SA signaling confer robustness to AvrRpt2-ETI

**Figure 7:**
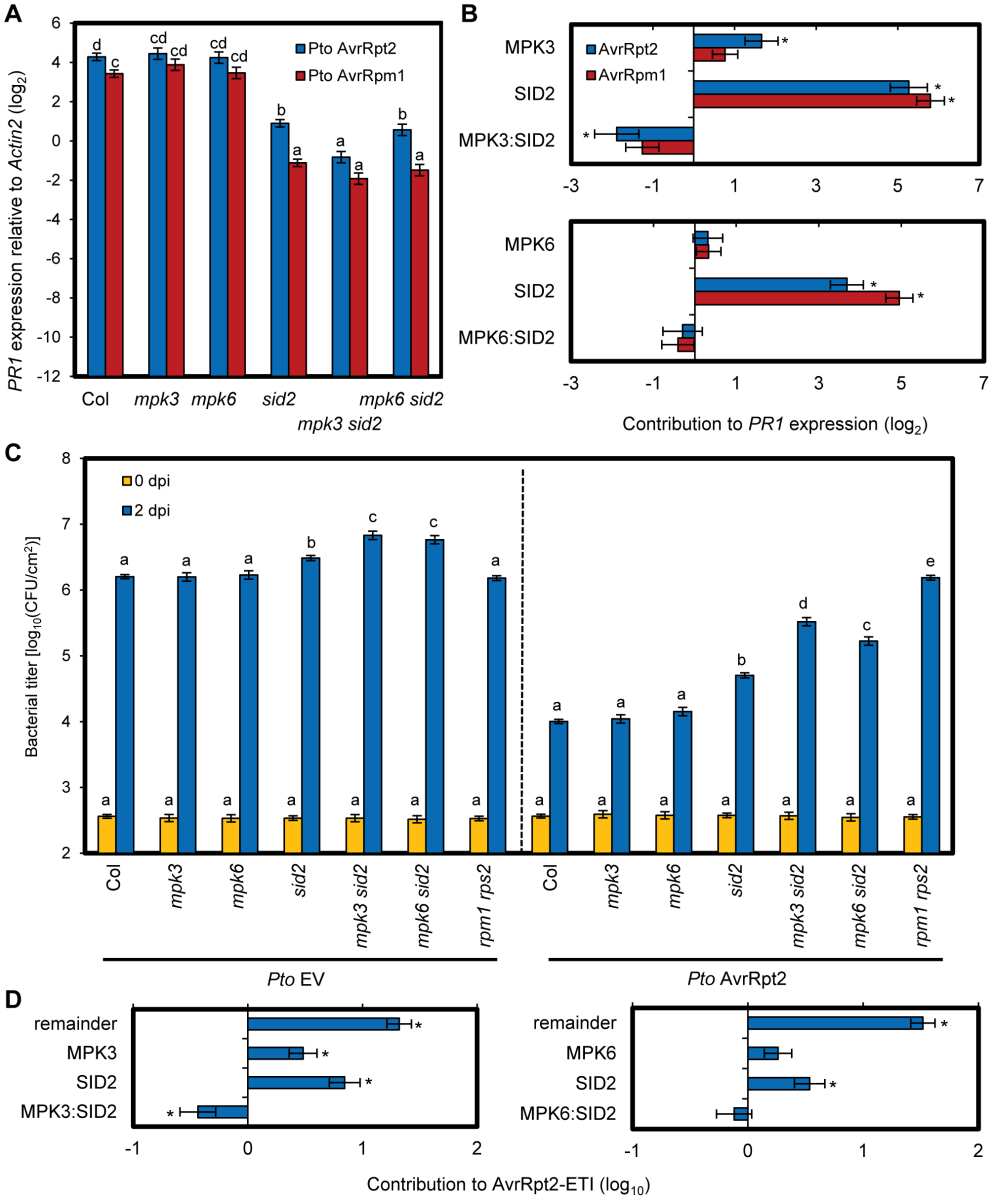
Compensation between MPK3 and SA contributes to the robust ETI levels. (A) The *PR1* expression level in leaves of the indicated genotypes at 24 hpi with *Pto* AvrRpt2 (blue bars) or AvrRpm1 (red bars) (OD_600_ = 0.001) was determined by qRT-PCR. Bars represent means and standard errors of three biological replicates calculated using a mixed linear model. The vertical axis shows the log_2_ expression level relative to *Actin2* (At2g18780). Statistically significant differences are indicated by different letters (*P*<0.01, two-tailed *t*-tests). (B) The signaling allocations for the *PR1* expression level shown in (A) were estimated for *MPK3* and *SID2* (upper panel) or *MPK6* and *SID2* (lower panel). (C) The bacterial counts of *Pto* EV (left panel) or AvrRpt2 (right panel) (inoculation dose, OD_600_ = 0.0001) at 0 or 2 dpi in leaves of the indicated genotypes were measured. Bars represent means and standard errors of three independent experiments with at least 4 or 12 biological replicates for 0 dpi or 2 dpi, respectively. Statistically significant differences are indicated by different letters per strain per dpi (*P*<0.01, two-tailed *t*-tests). (D) The signaling allocations for AvrRpt2-ETI shown in (C, 2 dpi) were estimated for *MPK3* and *SID2* (left panel) or *MPK6* and *SID2* (right panel). (B,D) Bars represent means and standard errors determined using a mixed linear model. Asterisks indicate significant effects or interaction (*P*<0.01).

We investigated if compensation between MPK3/MPK6 and SA signaling could be detected in the *PR1* expression level during ETI. Leaves of wild type (Col), *mpk3*, *mpk6*, *sid2*, *mpk3 sid2* and *mpk6 sid2* plants were inoculated with *Pto* AvrRpt2 or *Pto* AvrRpm1, and *PR1* expression levels were determined 24 hpi (Figure 7A). While *PR1* expression was compromised in *sid2* but not in *mpk3* or *mpk6* during AvrRpt2-ETI, it was compromised in *mpk3 sid2* more than in *sid2* (blue bar), suggesting compensation between *MPK3* and *SID2* on *PR1* expression during AvrRpt2-ETI. To quantify the level of compensation between *MPK3* and *SID2* on *PR1* expression, a signaling allocation analysis was applied [25]. In this analysis, the effects of the genes and their interactions were estimated for contribution to the *PR1* expression level after inoculation. We estimated the individual contribution of *MPK3* on the *PR1* expression level as the difference in expression levels between *sid2* and *mpk3 sid2*, that of *SID2* as the difference in *PR1* expression levels between *mpk3* and *mpk3 sid2* and their combined contribution as the difference in *PR1* expression levels between the wild type and *mpk3 sid2*. The value of the genetic interaction between *MPK3* and *SID2* was calculated by subtracting the sum of the individual contributions of *MPK3* and *SID2* from their combined contribution. Their combined contribution in the wild type was less than the sum of the individual contributions of SA and MPK3, which is signified by the negative interaction between them. We previously defined this less-than-additive combined contribution as compensation [25]. Such compensation was observed for AvrRpt2-ETI (Figure 7B, top). Thus, signaling mediated by MPK3 and SA is compensatory on *PR1* expression during AvrRpt2-ETI. No significant effects of *MPK6* or the interaction (*MPK6:SID2*) on *PR1* expression were detected during AvrRpt2-ETI (Figure 7A and B). No significant effects of *MPK3*, *MPK6* or their interactions (*MPK3:SID2* and *MPK6:SID2*) on *PR1* expression (Figure 7A and B, red bar) or resistance (Figure S11) were detected during AvrRpm1-ETI, suggesting a divergence in the mechanisms that modulate network robustness between different cases of ETI.

A similar trend was observed with the effects of MPK3 and MPK6 on bacterial resistance in AvrRpt2-ETI (Figure 7C). AvrRpt2-ETI is defined as the difference in *in planta* growth of *Pto* EV and *Pto* AvrRpt2 on a log_10_-scale [25]. The compensation between *MPK3* and *SID2* was clear from the signaling allocation analysis, as both had positive effects and their interaction was negative (Figure 7D, left). We did not detect significant effects of *MPK6* or the interaction (*MPK6:SID2*), although we observed a similar pattern to the case of *MPK3* (Figure 7D, right). Thus, compensation of SA signaling by a signaling mechanism involving MPK3 exists in inhibition of bacterial growth, as well as in *PR1* expression, during AvrRpt2-ETI.

Lethality of the double mutants *mpk3 mpk6* [21] does not allow us to determine combined contributions of MPK3 and MPK6 to compensation of SA signaling during ETI. It is possible that MPK6 is not a major factor in SA signaling compensation during ETI and that a signaling mechanism(s) other than that involving MPK3 or MPK6 is important during AvrRpm1-ETI. Nonetheless, these results clearly demonstrate that at least during AvrRpt2-ETI, SA signaling can be compensated by MPK3-mediated signaling in regulation of SA-responsive gene expression and that this compensation increases the robustness of the network output. This allows immunity to be maintained even if the major network sector, SA signaling, is compromised.

## Discussion

**Figure 8:**
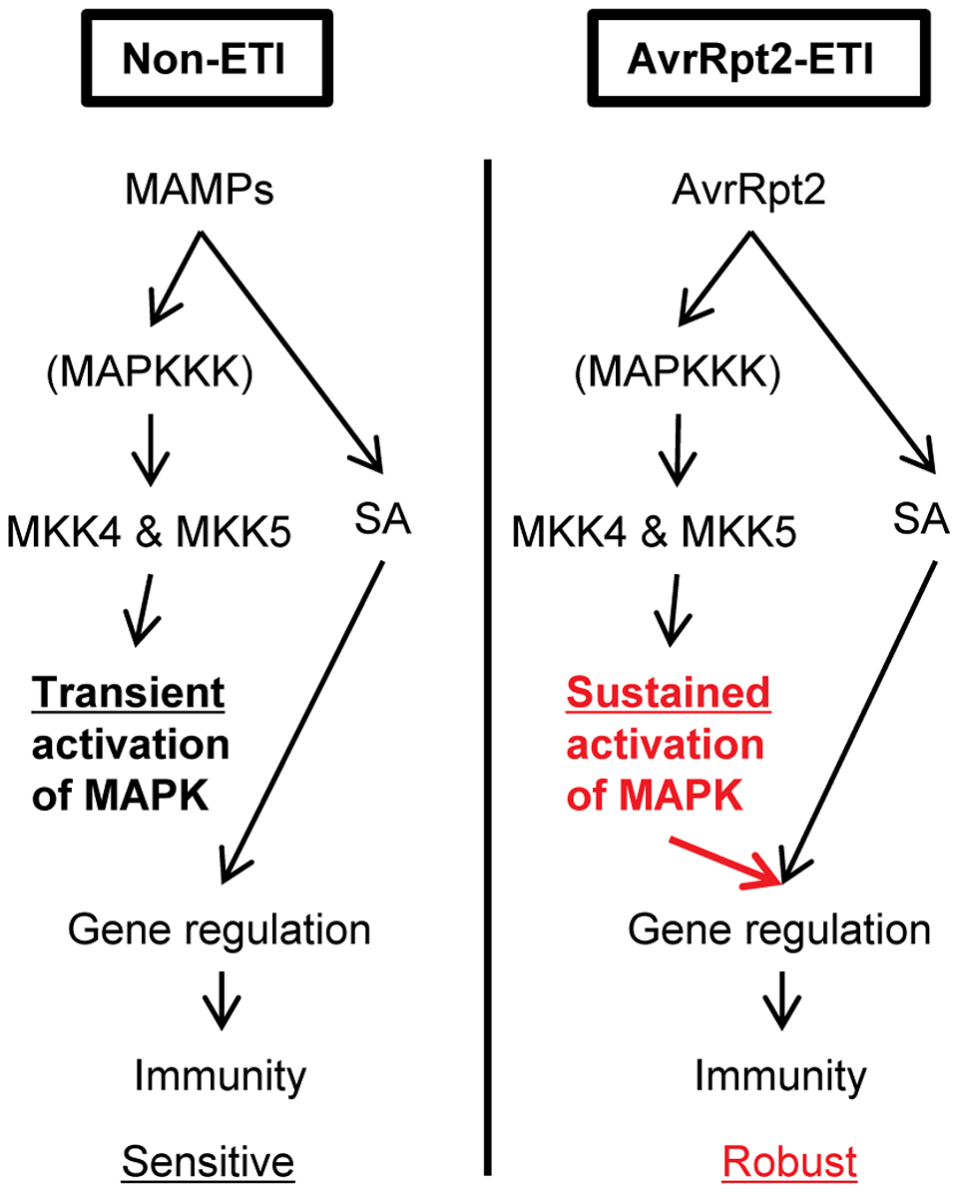
A model of signaling activated by sustained MAPK activation or SA signaling that regulates the common genes during AvrRpt2-ETI, resulting in robust immunity. During non-ETI, such as PTI, MAPK activation is transient. Transient MAPK activation is not sufficient for regulating the SA-responsive genes. However, during AvrRpt2-ETI, sustained MAPK activation can regulate the SA-responsive genes independently of SA. The differential duration of the MAPK activation can modulate the network property of robustness.

In this study, we identified a mechanism that can increase the robustness of the plant immune signaling network during AvrRpt2-ETI. Our results demonstrate that (1) MPK3 and MPK6 are activated in a sustained manner during ETI and in a transient manner during non-ETI; (2) Transient MAPK activation during non-ETI such as PTI does not contribute to SA-independent regulation of the SA-responsive genes; (3) Sustained MAPK activation activates an SA-independent alternative mechanism that regulates the SA-responsive genes; (4) SA-independent alternative mechanisms which regulate the SA-responsive genes were activated during AvrRpt2-ETI; (5) SA signaling compensation by the signaling sector involving MPK3 contributes to increased robustness against network perturbations during AvrRpt2-ETI (Figure 8).

### Factor(s) controlling differential activation duration of the MAPKs

A prior study implied that the duration of MPK3 and MPK6 activation is longer during ETI compared to during non-ETI upon *P. syringae* infection [27]. However, it did not rule out the possibility that the effector AvrRpt2 caused sustained MAPK activation through a mechanism independent of recognition of AvrRpt2 via RPS2. We clearly demonstrated that sustained MAPK activation occurs when ETI is triggered (Figures 3 and 4). The duration of MPK3 and/or MPK6 activation is the determinant for activation of the SA-independent alternative mechanism to regulate the SA-responsive genes: only sustained MAPK activation results in activation of the alternative mechanism. One potential cause of the differential activation duration is rapid turnover of PTI receptors, PRRs. FLS2 is rapidly degraded and disappears within one hour upon exposure to flg22 [33], [34]. Although turnover rates of other PRRs are not known, if many PRRs turn over rapidly upon activation, this could explain transient activation of the MAPKs by *Pto hrcC* (Figure 4), which presents multiple MAMPs [11]. The turnover rates of R proteins, the ETI receptors, upon their activation are largely unknown. Whether turnover rate is involved or not, this hypothesis that the duration of MAPK activation and, consequently, the robustness of the network can be tuned to each receptor is attractive because it would enable network robustness to be evolutionarily adapted according to what pathogen-derived signals are recognized by the receptors. Another potential but not mutually exclusive cause of the differential activation duration is involvement of protein phosphatases that dephosphorylate and inactivate the MAPKs: activation of the MAPKs may be negatively regulated by a phosphatase(s) during non-ETI responses while the phosphatase may be inactivated during ETI, resulting in the sustained activation of the MAPKs. Multiple types of such phosphatases including MAPK phosphatases are known in *Arabidopsis* [35]. Differential regulation of these phosphatases during ETI and non-ETI responses may explain the differential duration of MAPK activation.

### Decoding of the activation duration information

Switching of downstream signaling by differential duration of MAPK activation is known in animals and yeast [36]–[38]. In one case, it is explained by nuclear translocation of a MAPK that occurs only after its sustained activation [36]. In this way, sets of substrates available to the MAPK are distinct between its transient and sustained activation, which could lead to distinct downstream signaling. In plants, it has also been reported that MAPKs are translocated to the nucleus upon stimulation [39], [40]. Investigation of potential subcellular localization changes of *Arabidopsis* MPK3 and MPK6 during PTI and ETI will provide insight into this possibility. Another appealing explanation is involvement of a feed-forward network motif [41]. For example, activation of a transcription factor TF-X may mediate the alternative mechanism regulated by sustained MAPK activation. The activation of TF-X may require signal Y in addition to active MPK3 and/or MPK6. Signal Y may be slowly generated as a consequence of the activation of the MAPKs (e.g., 5 hours). The MAPKs would need to be activated for a long time to simultaneously have both signal Y and the active MAPKs to activate TF-X and regulate the SA-responsive genes. In either scenario, discovery of the signaling components downstream of the sustained MAPK activation will be the key to elucidate the mechanism that decodes duration of MAPK activation. Multiple transcription factors, such as TGAs, WRKYs, TBF1 and VIP1 [14], [22], [23], [42]–[44], are involved in regulation of *PR1*. These transcription factors may provide a good starting point for a search for the decoding mechanism.

### ETI may provide robustness under perturbation by coronatine

*Pto* produces the small molecule coronatine, which is a molecular mimic of the JA-Ile conjugate and promotes virulence by suppressing SA signaling [13]. *Pto* is highly virulent on *Arabidopsis* plants while ETI-triggering strains of *Pto*, such as *Pto* AvrRpt2, are much less virulent. Nevertheless, coronatine could suppress SA signaling. Therefore, SA-independent alternative mechanism(s) to regulate expression of the SA-responsive genes, such as that mediated by the MAPKs, may have a substantial role against perturbation of the immune signaling network by coronatine. This hypothesis is consistent with our observation that loss of both MPK3 and SA led to increased susceptibility to *Pto* AvrRpt2 (Figure 7).

### Can AvrRpt2-ETI overcome suppression effects by other effectors?

*Pto* DC3000 possesses type III effectors which directly or indirectly suppress MAPK activation [45]–[48]. However, we observed sustained activation of MPK3 and MPK6 during AvrRpt2-ETI when AvrRpt2 was delivered from *Pto* DC3000 (Figure 4). We speculate that the amounts of such MAPK-inhibiting type III effectors delivered and/or the kinetics of their delivery are not optimal to effectively suppress MAPK activation when the type III effectors are delivered from *Pto* DC3000, which represents a relatively natural context.

The effector HopAI1 from *Pto* DC3000 can physically interact with and inactivate MPK3 and MPK6 by removing the phosphate group from phosphothreonine via a phosphothreonine lyase activity [45]. HopAI1 also targets MPK4 and decreases MPK4 activity [48]. Decreased MPK4 activity appears to be monitored by the NB-LRR protein SUMM2, resulting in triggering ETI. Overexpression of HopAI1 in wild-type Col-0 plants but not *summ2* mutant plants leads to dwarfism and constitutive activation of immune responses [48]. However, *Pto* DC3000 does not trigger SUMM2-mediated ETI. Consistently, *HopAI1* of *Pto* DC3000 is disrupted by an insertion in its promoter region [49]. Thus, the amount of HopAI1 delivered from *Pto* DC3000 appears insufficient for effective inhibition of MPK3 and MPK6 activation during AvrRpt2-ETI.

Another effector, HopF2, from *Pto* DC3000 can also suppress activity of MPK3, MPK4 and MPK6 by targeting the upstream MKK5 and likely other MKKs as well [46], [47]. When overexpressed in plants, HopF2 interferes with AvrRpt2-ETI by inhibiting AvrRpt2-mediated RIN4 degradation [50]. Again, the reason that HopF2 cannot suppress sustained activation of MPK3 and MPK6 triggered by AvrRpt2 when it is delivered from *Pto* DC3000 (Figure 4) is likely insufficient HopF2 or inappropriate timing of its delivery. Delivery of AvrRpt2 may precede that of HopF2 [50].

### Is the low level of robustness required during PTI?

One enigma is why plants need to make the robustness of the immune signaling network lower during PTI when the network itself has the capacity to be highly robust. If the network output during PTI were as robust as during ETI, the chance that “true” pathogens will overcome PTI would be much lower. We speculate that the lower robustness during PTI is selected through evolution as trade-offs with other requirements. Many MAMPs are shared among pathogens and benign microbes and provide low quality information about pathogen attack. It is probably not adaptive for plants to respond to a MAMP with strong and sustained immune responses similar to those during ETI since in many cases, plants encounter benign microbes and ETI-type responses cost fitness. A strategy apparently selected is to respond weakly first and wait to intensify the response until further information increases the probability that a true pathogen is present [24]. In contrast, since effectors are a hallmark of true pathogens and provide high quality information, during ETI plants can induce rapid and strong immune responses with a very low chance of needless fitness costs.

### Concluding remarks

The signaling sector activated by sustained activation of the MAPKs during ETI and the SA signaling sector can regulate the common set of genes. This is one of the mechanisms underlying robustness of the immunity level against network perturbations during ETI. This modulation of the network robustness is controlled by signaling kinetics of a network component. Our findings imply that properties of biological networks can be modulated through network component activities.

## Materials and Methods

Free SA measurement, MAP kinase assays, bacterial growth assays and the signaling allocation analysis were performed as described previously [25], [26].

### Plant materials and growth conditions

*Arabidopsis* plants were grown in a controlled environment at 22°C with a 12 h photoperiod and 75% relative humidity. *Arabidopsis thaliana* accession Col-0 was the background of all mutants used in this study. *Arabidopsis mpk3-1* (SALK_151594) [21], *mpk6-2* (SALK_073907) [18], *npr1-1* [51], *rps2* 101C [52] and *sid2-2* [15] were previously described. We generated the double mutants *mpk3 sid2* and *mpk6 sid2* by standard genetic crosses. Estradiol-inducible AvrRpt2 transgenic lines [53] and the *DEX-MKK4DD* and -*MKK5DD* transgenic lines [30] were previously described. We crossed *DEX-MKK4DD* and *-MKK5DD* into the mutant backgrounds *mpk3*, *mpk6*, *npr1*, *sid2* and *vip1*. Primers and restriction enzymes used for screening of the mutants are listed in Table S4.

### Chemicals

Flg22 peptide was purchased from EZBiolab Inc (Westfield, IN, USA). Estradiol (E8875) and DEX (D1756) were purchased from Sigma (Saint Louis, MO, USA).

### Quantitative RT-PCR analysis

*Pto* DC3000 strains (or water for mock) or 2 µM DEX (or 0.1% ethanol for mock) were infiltrated into leaves of 4-week-old plants. Leaves were collected at the indicated time points. Total RNA isolation and qRT-PCR analysis were carried out as described previously [54], [55]. The following models were fit to the relative Ct value data compared to *Actin2* using the lme function in the nlme package in the R environment: *C_tgytr_* = *GYT_gyt_*+*R_r_*+*ε_gytr_*, where *GYT*, genotype:treatment:time interaction, and random factors; *R*, biological replicate; *ε*, residual; *C_tgyr_* = *GY_gy_*+*R_r_*+*ε_gytr_*, where *GY*, genotype:treatment interaction; *C_tgtr_* = *GT_gt_*+*R_r_*+*ε_gtr_*, where *GT*, genotype:time interaction. The mean estimates of the fixed effects were used as the modeled relative Ct values and visualized as the relative log_2_ expression values and compared by two-tailed *t*-tests. For the *t*-tests, the standard errors were calculated using the variance and covariance values obtained from the model fitting. Primers used in the study are listed in Table S4.

### DNA microarrays

Four-week-old *Arabidopsis* Col-0 and *sid2* leaves were infiltrated with *Pto hrcC*, *Pto* pLAFR (EV), *Pto* AvrRpt2 or water (mock). Independently, leaves of four-week-old *DEX-MKK4DD* plants in Col-0 or a *sid2* background were infiltrated with 2 µM DEX or 0.1% ethanol (mock). Samples were collected at 24 hpi. Total RNA was extracted as described previously [26] and profiled using the NimbleGen DNA microarray (*A. thaliana* Gene Expression 12×135K array TAIR9.0) following the manufacturer's protocol (Roche Applied Science, Indianapolis, IN, USA). Three independent experiments (biological replicates) were performed. The microarray data were submitted to Gene Expression Omnibus (Accession, GSE40555). Probe signal values were subjected to the robust multi-array average (RMA) summarization algorithm [56] using the standard NimbleGen software to obtain the expression level values of the transcripts. Among transcripts of a single gene, those with higher expression values were selected as the representative transcripts of the genes. The following models were fit to log_2_ expression values using the lmFit function in the limma package in the R environment: *S_gyr_* = *GY_gyt_*+*R_r_*+*ε_gyr_*, where *S*, log2 expression value, *GY*, genotype:treatment interaction, and random factors; *R*, biological replicate; *ε*, residual. The eBayes function in the limma package was used for variance shrinkage in calculation of the *p*-values and the Storey's *q*-values were calculated from the *p*-values using the qvalue function in the qvalue package. First, genes whose expression was up-regulated or down-regulated (*q* values<0.01 and more than 2 fold change) in both *Pto* EV and *Pto* AvrRpt2-infected Col compared to mock were selected (2828 genes). Second, *SID2*-dependent genes in *Pto* EV infection (inductions/suppression in *sid2* are less than 20% compared to Col) were selected (187 “SA-responsive” genes) for the clustering analysis. Heatmaps were generated by CLUSTER [57] using uncentered Pearson correlation and complete linkage, and visualized by TREEVIEW [57].

### Accession numbers

The accession numbers for the *Arabidopsis* genes discussed in this article are as follows: *Actin2* (At2g18780), *Chitinase* (At1g02360), *CHS* (At5g13930), *FRK1* (At2g19190), *MKK4* (At1g51660), *MKK5* (At3g21220), *MPK3* (At3g45640), *MPK4* (At4g01370), *MPK6* (At2g43790), *NPR1* (At1g64280), *RPM1* (At3g07040), *RPS2* (At4g26090) and *SID2* (At1g74710).

## Supporting Information

Figure S1 *PR1* induction during ETI is largely NPR1-independent. The *PR1* expression level in leaves inoculated with *Pto* strains (OD_600_ = 0.001) or mock was determined by qRT-PCR at 24 hpi. Bars represent means and standard errors of two biological replicates calculated using a mixed linear model. The vertical axis shows the log_2_ expression level relative to *Actin2* (At2g18780). Asterisks indicate significant differences from mock (*P*<0.01, two-tailed *t*-tests).
(TIF)

Figure S2 Expression patterns after inoculation with *Pto hrcC* and *Pto* EV are similar while that with *Pto* AvrRpt2 is distinctive in terms of SID2-dependency. (A) A heatmap of pathogen-regulated genes. Leaves were collected at 24 hpi with the indicated *Pto* strains (OD_600_ = 0.001) or mock and mRNA profile analysis was performed using a NimbleGen Array. Genes whose expression was up- or down-regulated (*q* values<0.01 and more than 2 fold change) in any samples compared to mock were selected (5361 genes). The log_2_ ratios compared to mock for the selected genes were subjected to agglomerative hierarchical clustering analysis. Green indicates negative values, red indicates positive values and black indicates zero. (B) A normalized heatmap. Expression changes after inoculation with *Pto hrcC*, *Pto* EV and *Pto* AvrRpt2 were compared using linear regression. Based on the regression coefficients, the log_2_ ratios of *Pto hrcC* and *Pto* AvrRpt2 samples were weighted by factors of 1.98 and 0.76, respectively, to normalize the overall level of induction/suppression among the treatments.
(TIF)

Figure S3 *PR1* induction is independent of SA during AvrRpt2-ETI. Seedlings of transgenic lines carrying the estradiol-inducible *AvrRpt2* transgene in a *sid2* background (*XVE-AvrRpt2*/*sid2*) were treated with different concentrations of estradiol for 24 hours in a liquid medium. The *PR1* expression level was determined by qRT-PCR. Bars represent means and standard errors of two biological replicates calculated using a mixed linear model. The vertical axis shows the log_2_ expression level relative to *Actin2* (At2g18780). Asterisks indicate significant differences from mock (*P*<0.01, two-tailed *t*-tests).
(TIF)

Figure S4 The MAPKs activated in a sustained manner during AvrRpt2- and AvrRps4-ETI were MPK3 and MPK6. Leaves of Col, *mpk3* and *mpk6* plants were infiltrated with *Pto* AvrRpt2 (A) or *Pto* AvrRps4 (B) (OD_600_ = 0.01) and samples were collected at the indicated time points. Activated MAPKs were detected by immunoblot using anti-p44/42 MAPK antibody. Ponceau S stained blots are shown for loading controls. Experiments were conducted twice with similar results.
(TIF)

Figure S5 Sustained MAPK activation is independent of SA. Leaves of Col and *sid2* plants were infiltrated with *Pto* EV (A) or *Pto* AvrRpt2 (B) at an inoculation dose of OD_600_ = 0.01 and samples were collected at the indicated time points. Activated MAPKs were detected by immunoblot using anti-p44/42 MAPK antibody. Ponceau S stained blots are shown as loading controls. Experiments were conducted twice with similar results.
(TIF)

Figure S6 Sustained MAPK activation by MKK4DD and MKK5DD expression. Leaves of *DEX-MKK4DD* (MKK4DD) or *-MKK5DD* (MKK5DD) were treated with 2 µM DEX for the indicated times and activated MAPKs were detected by immunoblot using anti-p44/42 MAPK antibody. Ponceau S stained blots are shown as loading controls. Experiments were conducted twice with similar results.
(TIF)

Figure S7 Induction of *FRK1* by flg22 does not require SA. Leaves of Col or *sid2* plants were infiltrated with 1 µM flg22 or water and samples were collected at the indicated time points. The expression level of *FRK1* was determined by qRT-PCR. Bars represent means and standard errors of at least two biological replicates calculated using a mixed linear model. The vertical axis is the log_2_ expression level relative to *Actin2* (At2g18780). Asterisks indicate significant differences from mock (*P*<0.01, two-tailed *t*-tests).
(TIF)

Figure S8 MPK3 seems to be required for SA-independent *PR1* induction conferred by forced MKK5 activation while MPK6 is dispensable. Leaves of transgenic plants carrying DEX-inducible MKK4DD (4DD) or MKK5DD (5DD) (Col, *mpk3* or *mpk6* background) were infiltrated with 2 µM DEX (DEX) or 0.1% ethanol (mock) and samples were collected at 24 hpi. The expression levels of *PR1* (A) and *MKK4* or *MKK5* (B) were determined by qRT-PCR. Bars represent means and standard errors of two biological replicates calculated using a mixed linear model. The vertical axis is the log_2_ expression level relative to *Actin2* (At2g18780). Asterisks indicate significant differences from mock (*P*<0.01, two-tailed *t*-tests).
(TIF)

Figure S9 Expression patterns in DEX-inducible *MKK4DD* transgenic plants are very similar in Col and *sid2*. Leaves of transgenic plants carrying DEX-inducible MKK4DD in Col or *sid2* backgrounds were infiltrated with 2 µM DEX or 0.1% ethanol and samples were collected at 24 hpi. mRNA profile analysis was performed as described in Figure 2. Genes whose expression was up-regulated or down-regulated (*q* values<0.01 and more than 4 fold change) in DEX-treated samples compared to mock were selected (4743 genes). The log_2_ ratios (DEX/mock) for the 4743 genes were subjected to agglomerative hierarchical clustering analysis as in Figure 2. Green indicates negative values, red indicates positive values and black indicates zero.
(TIF)

Figure S10 Expression patterns of genes representing three clusters. Leaves of Col or *sid2* plants were infiltrated with *Pto* EV, *Pto* AvrRpt2 (OD_600_ = 0.001) or water (mock) and samples were collected at 24 hpi. Leaves of transgenic plants carrying DEX-inducible AtMKK4DD in a *sid2* background were infiltrated with 2 µM DEX or 0.1% ethanol and samples were collected at 24 hpi. The expression levels of *PR1* (At2g14610) (A), *Chitinase* (At1g02360) (B) and *CHS* (At5g13930) (C), which represent Clusters I, II, and III, respectively, were determined by qRT-PCR. Bars represent means and standard errors of three biological replicates calculated using a mixed linear model. The vertical axis is the log_2_ expression level relative to *Actin2* (At2g18780). Asterisks indicate significant differences from mock (*P*<0.01, two-tailed *t*-tests).
(TIF)

Figure S11 AvrRpm1-ETI. (A) The bacterial counts of *Pto* EV (left panel) or AvrRpm1 (right panel) (inoculation dose, OD_600_ = 0.0001) at 0 or 2 dpi in leaves of the indicated genotypes were measured. Bars represent means and standard errors of three independent experiments with at least 4 or 12 biological replicates for 0 dpi or 2 dpi, respectively. Statistically significant differences are indicated by different letters per strain per dpi (*P*<0.01, two-tailed *t*-tests). (B) The signaling allocations for AvrRpm1-ETI shown in (A, 2 dpi) were estimated for *MPK3* and *SID2* (left panel) or *MPK6* and *SID2* (right panel). Bars represent means and standard errors determined using a mixed linear model. Asterisks indicate significant effects or interaction (*P*<0.01).
(TIF)

Table S1 Expression levels of the SA-responsive 187 genes and cluster information in Figure 2.
(XLSX)

Table S2 Expression levels of the genes in Figure S2.
(XLSX)

Table S3 Expression levels of the genes in Figure S9.
(XLSX)

Table S4 Primers used in this study.
(DOCX)
