# Supplementary figures and images for: Dual Regulation of Gene Expression Mediated by Extended MAPK Activation and Salicylic Acid Contributes to Robust Innate Immunity in Arabidopsis thaliana
Source: PLoS Genet. 2013 Dec 12;9(12):e1004015. doi: 10.1371/journal.pgen.1004015 (PMC3861249; doi:10.1371/journal.pgen.1004015)

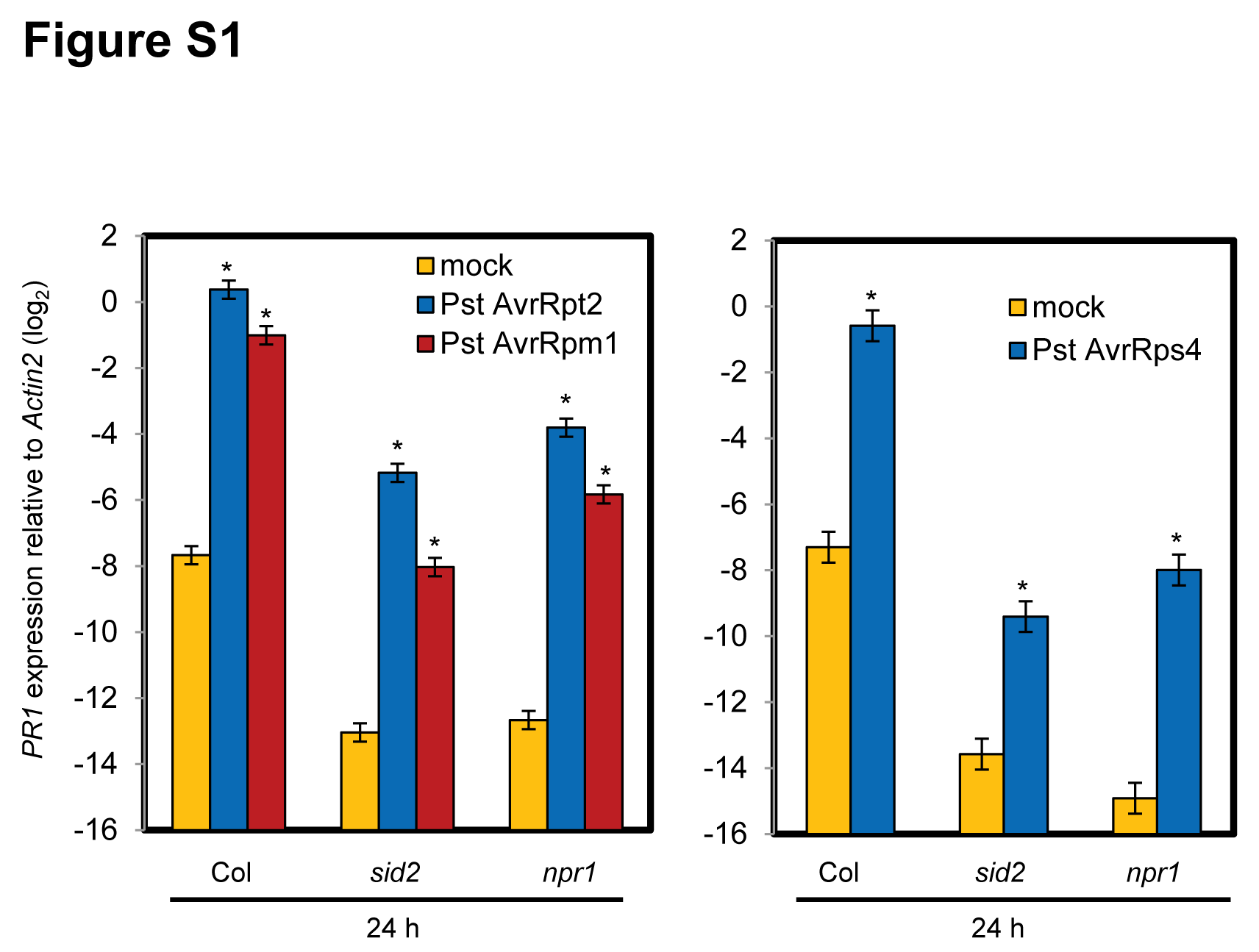

Supplement: Figure S1 — PR1 induction during ETI is largely NPR1-independent. The PR1 expression level in leaves inoculated with Pto strains (OD600 = 0.001) or mock was determined by qRT-PCR at 24 hpi. Bars represent means and standard errors of two biological replicates calculated using a mixed linear model. The vertical axis shows the log2 expression level relative to Actin2 (At2g18780). Asterisks indicate significant differences from mock (P<0.01, two-tailed t-tests). (TIF) [file pgen.1004015.s001.tif]

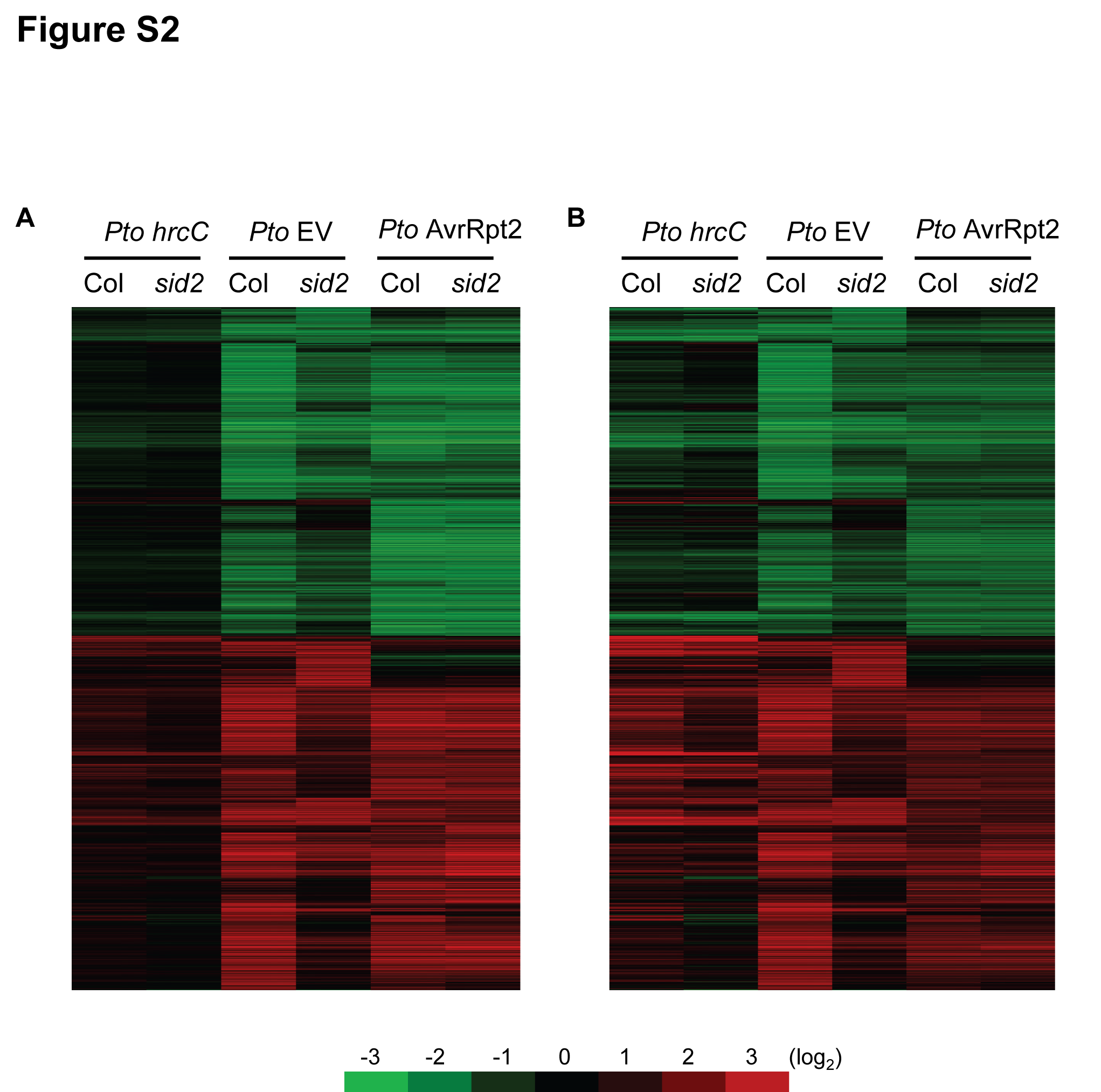

Supplement: Figure S2 — Expression patterns after inoculation with Pto hrcC and Pto EV are similar while that with Pto AvrRpt2 is distinctive in terms of SID2-dependency. (A) A heatmap of pathogen-regulated genes. Leaves were collected at 24 hpi with the indicated Pto strains (OD600 = 0.001) or mock and mRNA profile analysis was performed using a NimbleGen Array. Genes whose expression was up- or down-regulated (q values<0.01 and more than 2 fold change) in any samples compared to mock were selected (5361 genes). The log2 ratios compared to mock for the selected genes were subjected to agglomerative hierarchical clustering analysis. Green indicates negative values, red indicates positive values and black indicates zero. (B) A normalized heatmap. Expression changes after inoculation with Pto hrcC, Pto EV and Pto AvrRpt2 were compared using linear regression. Based on the regression coefficients, the log2 ratios of Pto hrcC and Pto AvrRpt2 samples were weighted by factors of 1.98 and 0.76, respectively, to normalize the overall level of induction/suppression among the treatments. (TIF) [file pgen.1004015.s002.tif]

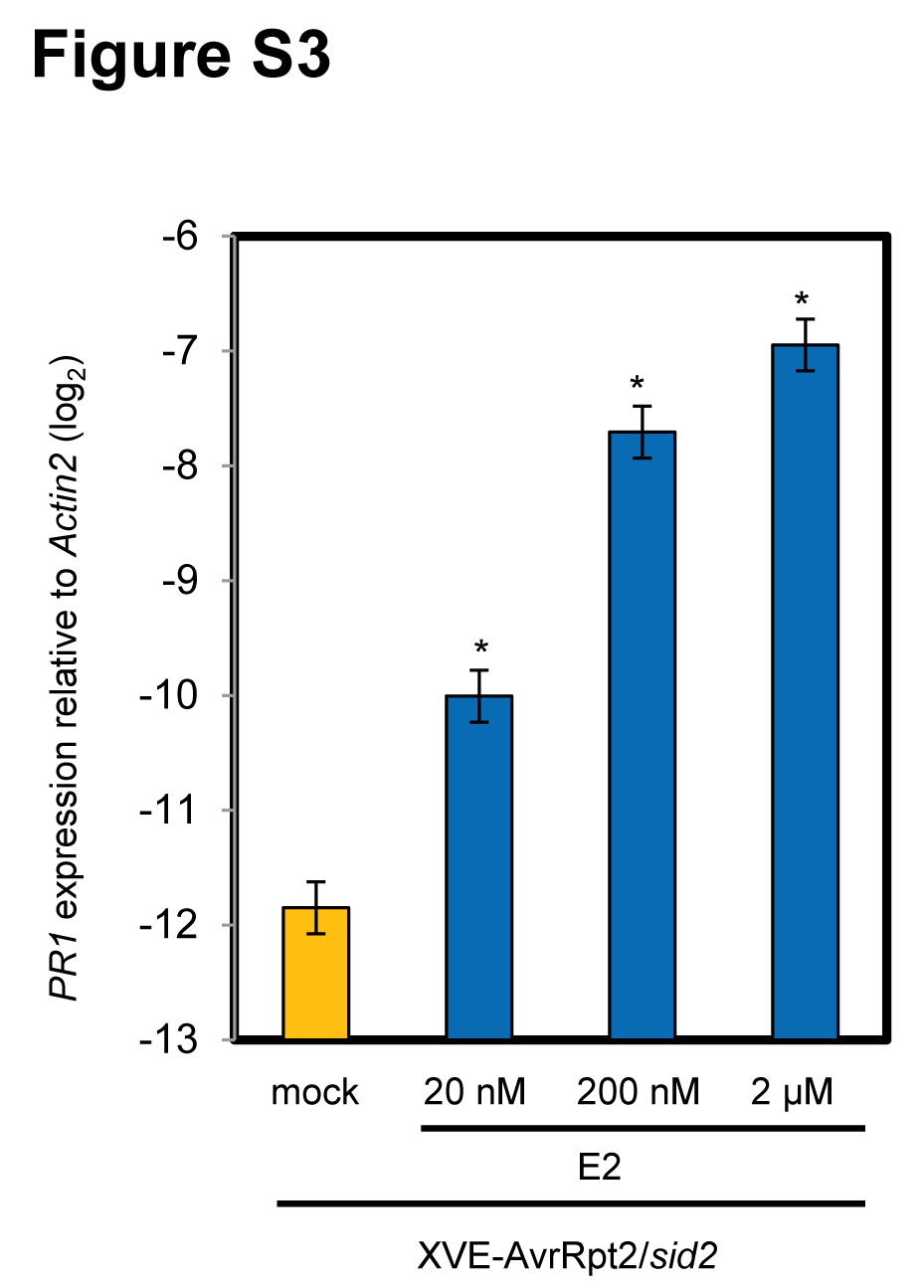

Supplement: Figure S3 — PR1 induction is independent of SA during AvrRpt2-ETI. Seedlings of transgenic lines carrying the estradiol-inducible AvrRpt2 transgene in a sid2 background (XVE-AvrRpt2/sid2) were treated with different concentrations of estradiol for 24 hours in a liquid medium. The PR1 expression level was determined by qRT-PCR. Bars represent means and standard errors of two biological replicates calculated using a mixed linear model. The vertical axis shows the log2 expression level relative to Actin2 (At2g18780). Asterisks indicate significant differences from mock (P<0.01, two-tailed t-tests). (TIF) [file pgen.1004015.s003.tif]

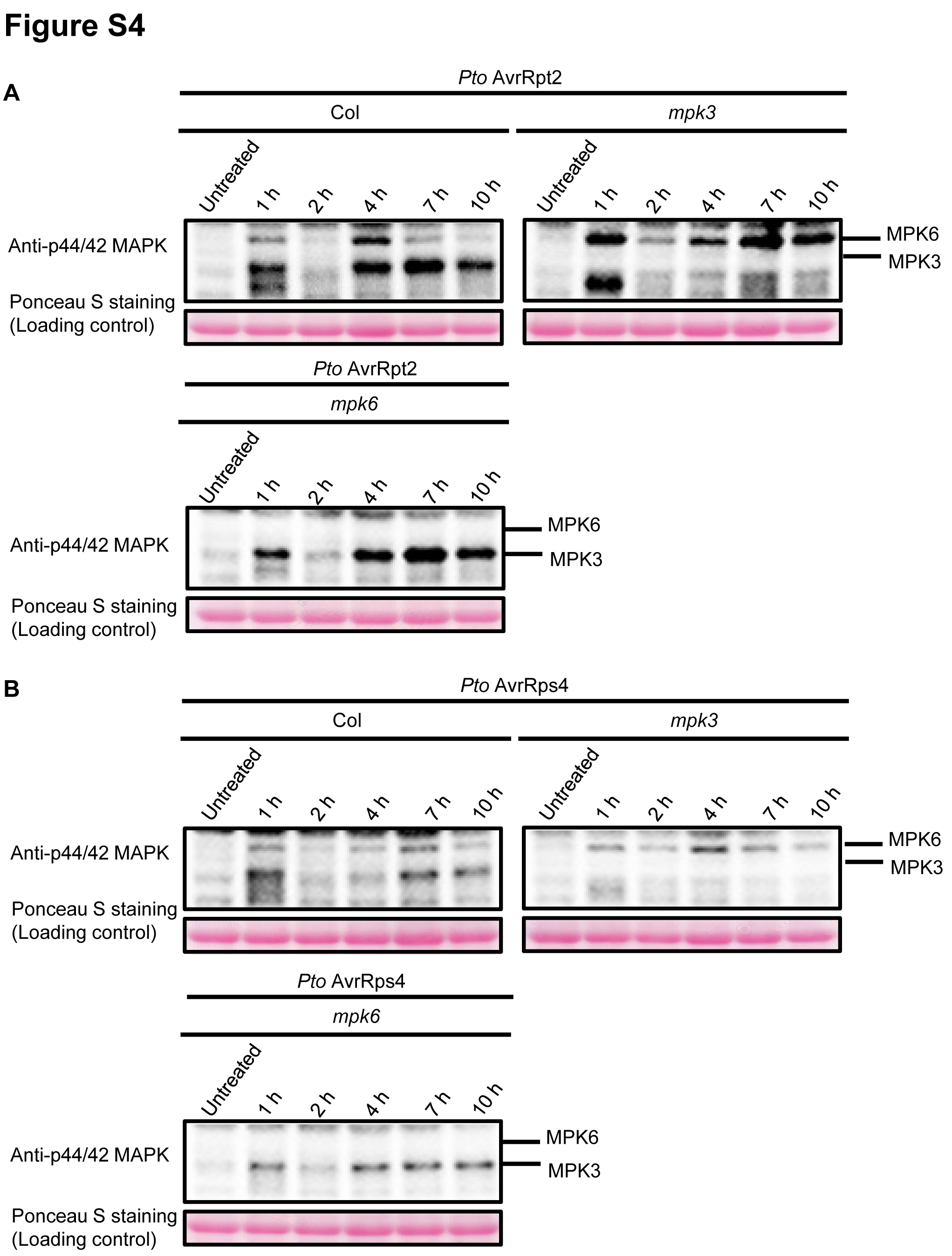

Supplement: Figure S4 — The MAPKs activated in a sustained manner during AvrRpt2- and AvrRps4-ETI were MPK3 and MPK6. Leaves of Col, mpk3 and mpk6 plants were infiltrated with Pto AvrRpt2 (A) or Pto AvrRps4 (B) (OD600 = 0.01) and samples were collected at the indicated time points. Activated MAPKs were detected by immunoblot using anti-p44/42 MAPK antibody. Ponceau S stained blots are shown for loading controls. Experiments were conducted twice with similar results. (TIF) [file pgen.1004015.s004.tif]

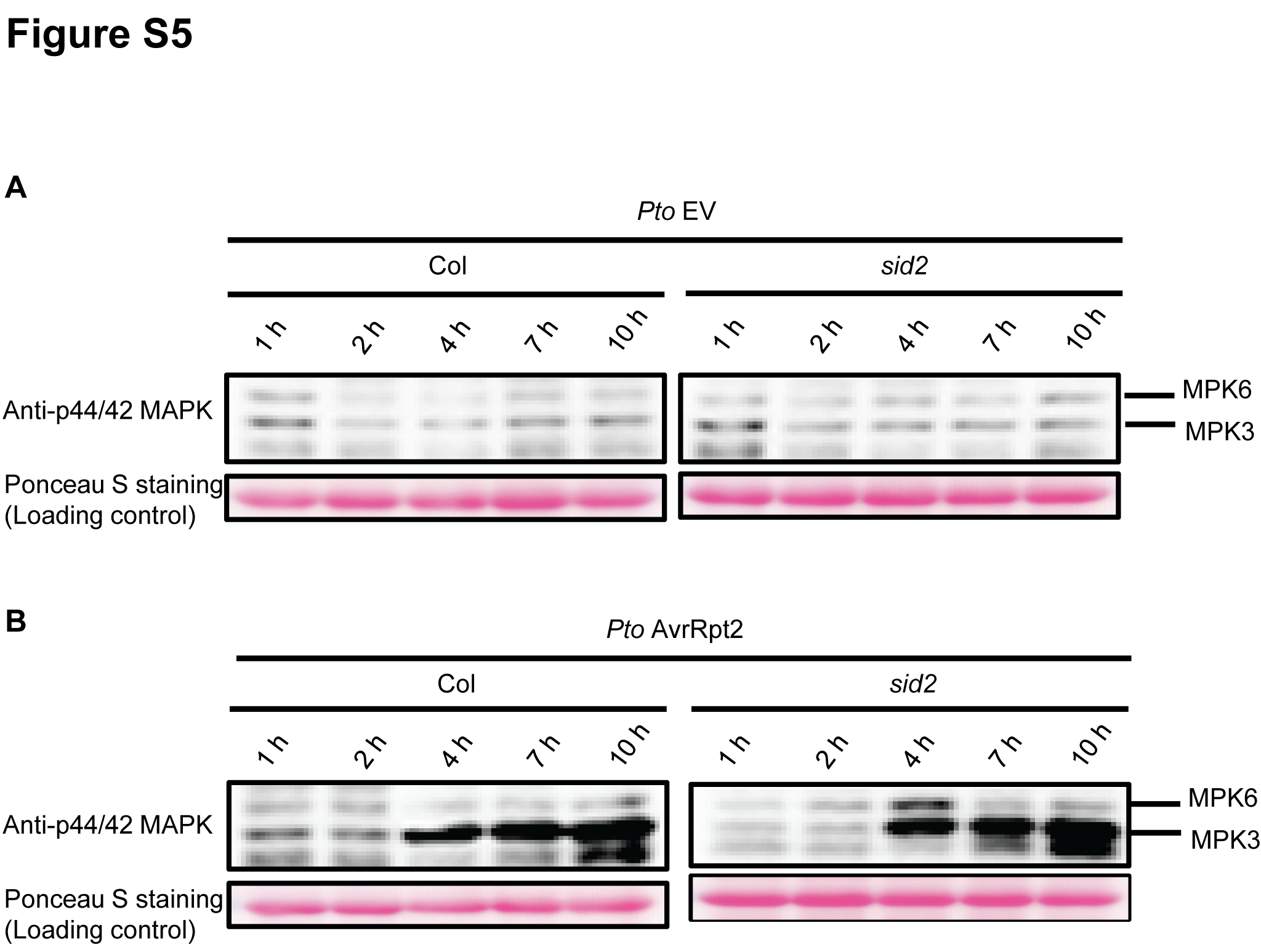

Supplement: Figure S5 — Sustained MAPK activation is independent of SA. Leaves of Col and sid2 plants were infiltrated with Pto EV (A) or Pto AvrRpt2 (B) at an inoculation dose of OD600 = 0.01 and samples were collected at the indicated time points. Activated MAPKs were detected by immunoblot using anti-p44/42 MAPK antibody. Ponceau S stained blots are shown as loading controls. Experiments were conducted twice with similar results. (TIF) [file pgen.1004015.s005.tif]

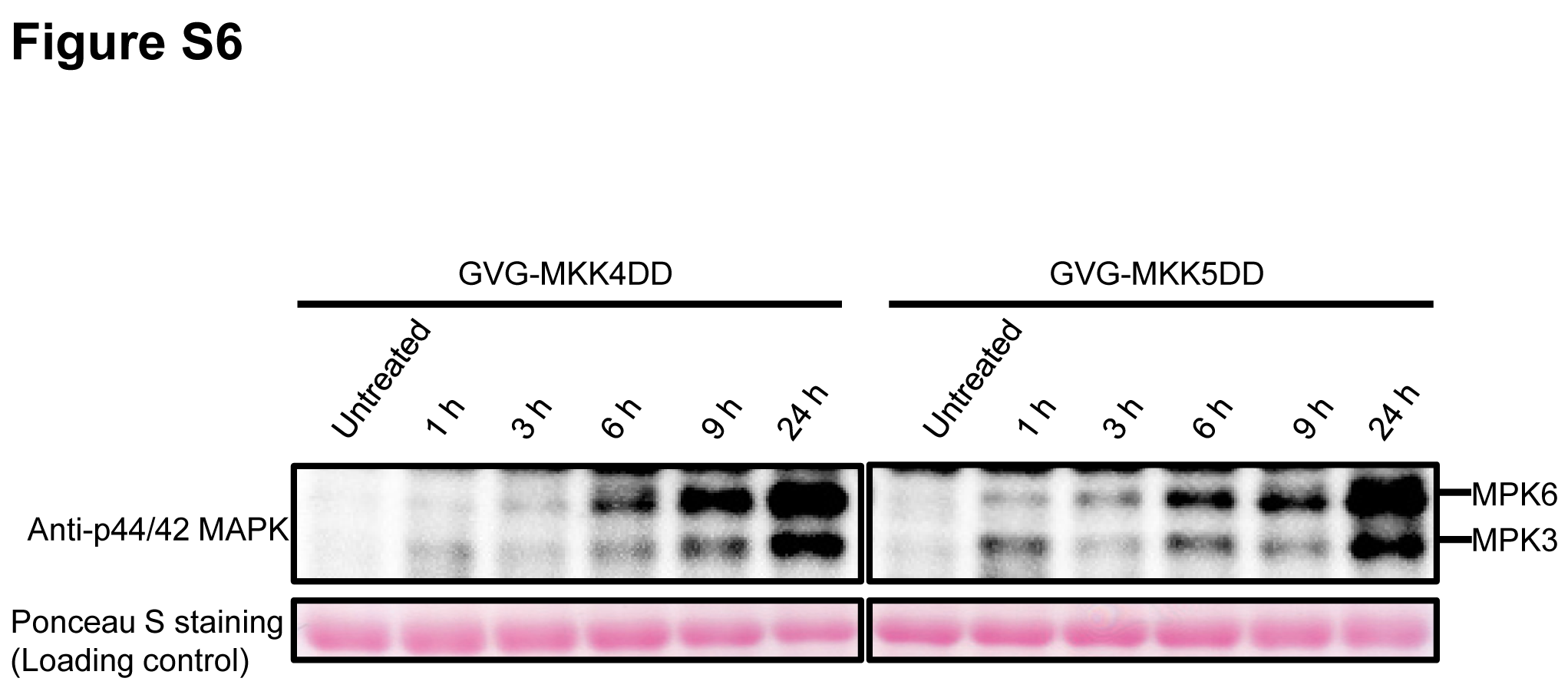

Supplement: Figure S6 — Sustained MAPK activation by MKK4DD and MKK5DD expression. Leaves of DEX-MKK4DD (MKK4DD) or -MKK5DD (MKK5DD) were treated with 2 µM DEX for the indicated times and activated MAPKs were detected by immunoblot using anti-p44/42 MAPK antibody. Ponceau S stained blots are shown as loading controls. Experiments were conducted twice with similar results. (TIF) [file pgen.1004015.s006.tif]

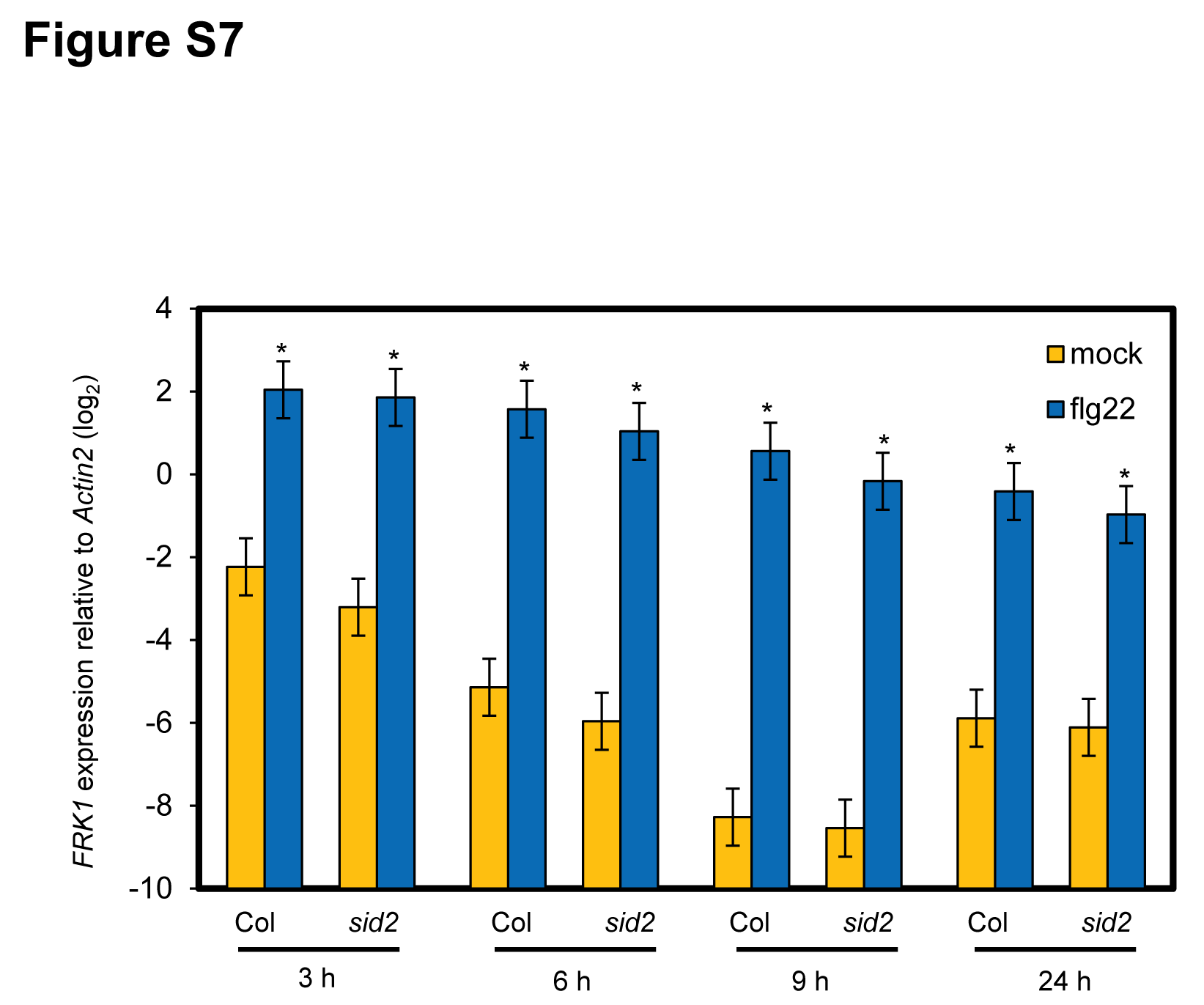

Supplement: Figure S7 — Induction of FRK1 by flg22 does not require SA. Leaves of Col or sid2 plants were infiltrated with 1 µM flg22 or water and samples were collected at the indicated time points. The expression level of FRK1 was determined by qRT-PCR. Bars represent means and standard errors of at least two biological replicates calculated using a mixed linear model. The vertical axis is the log2 expression level relative to Actin2 (At2g18780). Asterisks indicate significant differences from mock (P<0.01, two-tailed t-tests). (TIF) [file pgen.1004015.s007.tif]

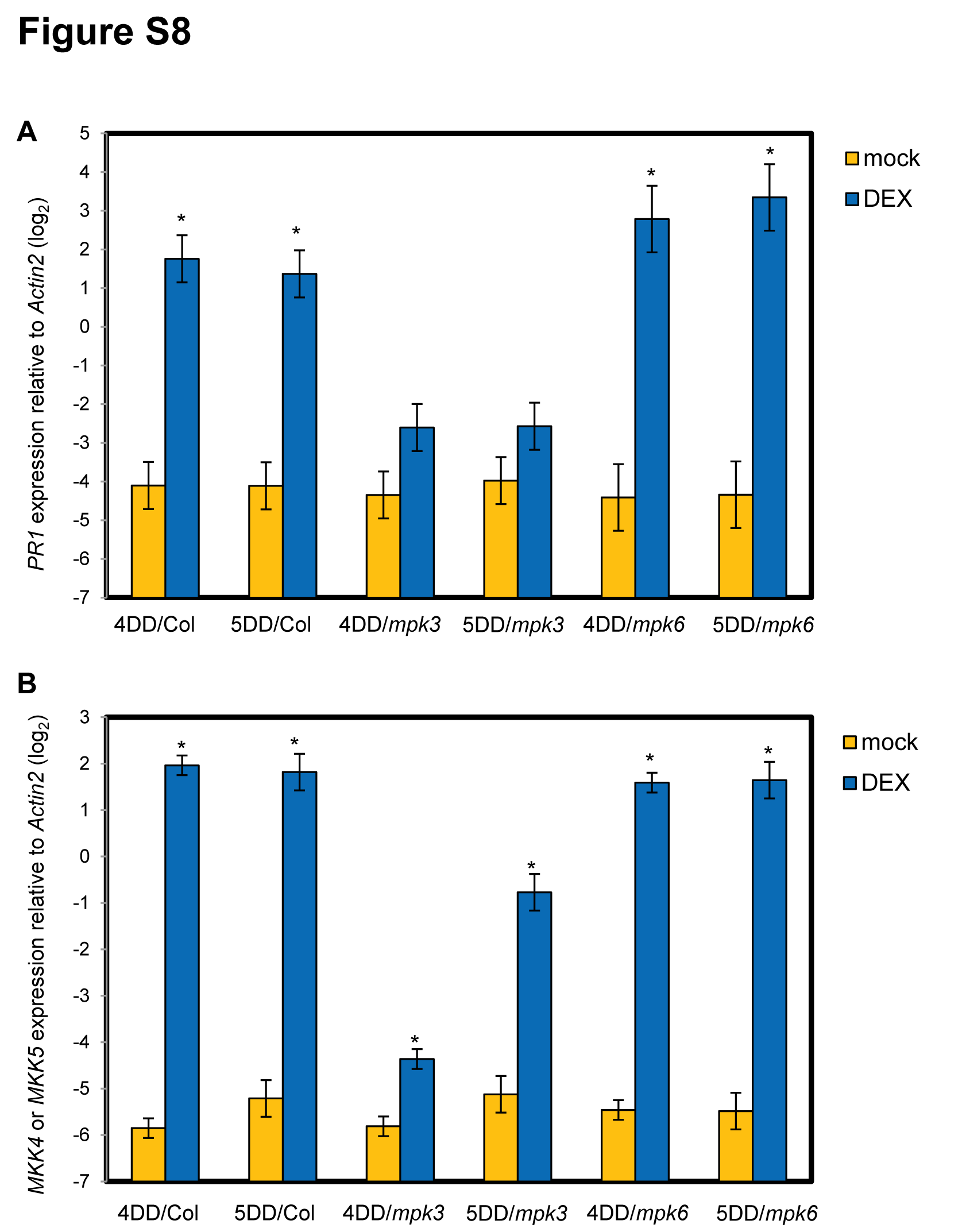

Supplement: Figure S8 — MPK3 seems to be required for SA-independent PR1 induction conferred by forced MKK5 activation while MPK6 is dispensable. Leaves of transgenic plants carrying DEX-inducible MKK4DD (4DD) or MKK5DD (5DD) (Col, mpk3 or mpk6 background) were infiltrated with 2 µM DEX (DEX) or 0.1% ethanol (mock) and samples were collected at 24 hpi. The expression levels of PR1 (A) and MKK4 or MKK5 (B) were determined by qRT-PCR. Bars represent means and standard errors of two biological replicates calculated using a mixed linear model. The vertical axis is the log2 expression level relative to Actin2 (At2g18780). Asterisks indicate significant differences from mock (P<0.01, two-tailed t-tests). (TIF) [file pgen.1004015.s008.tif]

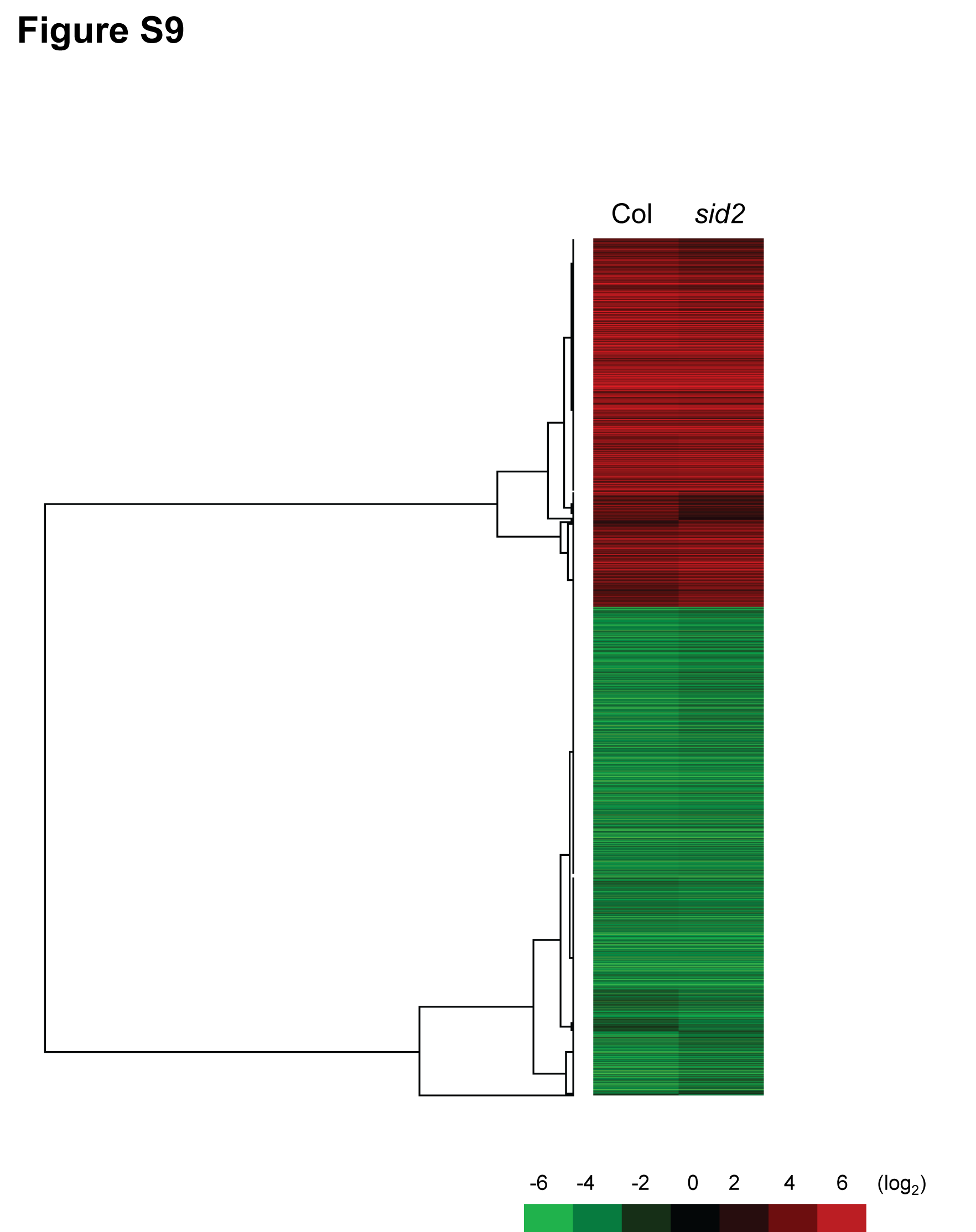

Supplement: Figure S9 — Expression patterns in DEX-inducible MKK4DD transgenic plants are very similar in Col and sid2. Leaves of transgenic plants carrying DEX-inducible MKK4DD in Col or sid2 backgrounds were infiltrated with 2 µM DEX or 0.1% ethanol and samples were collected at 24 hpi. mRNA profile analysis was performed as described in Figure 2. Genes whose expression was up-regulated or down-regulated (q values<0.01 and more than 4 fold change) in DEX-treated samples compared to mock were selected (4743 genes). The log2 ratios (DEX/mock) for the 4743 genes were subjected to agglomerative hierarchical clustering analysis as in Figure 2. Green indicates negative values, red indicates positive values and black indicates zero. (TIF) [file pgen.1004015.s009.tif]

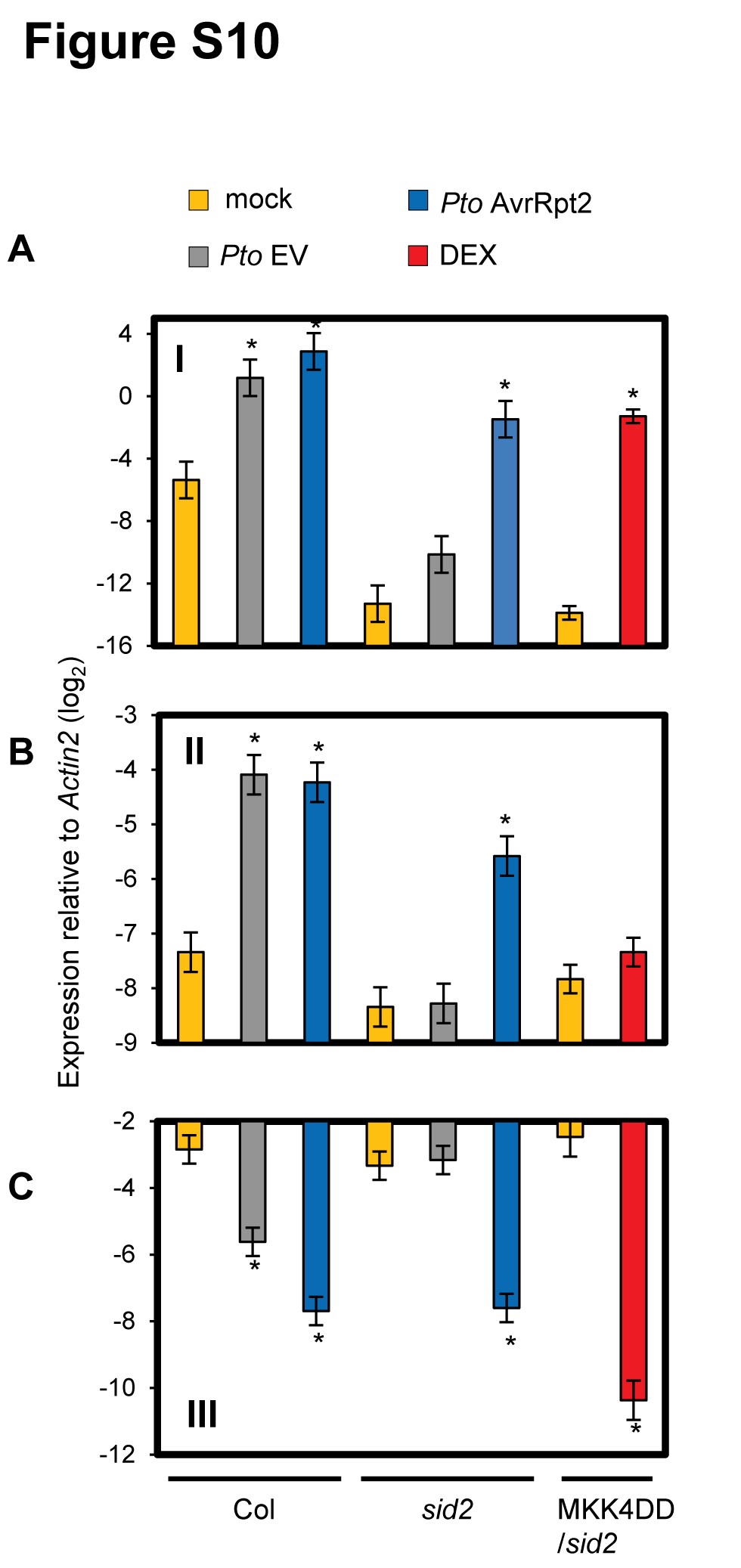

Supplement: Figure S10 — Expression patterns of genes representing three clusters. Leaves of Col or sid2 plants were infiltrated with Pto EV, Pto AvrRpt2 (OD600 = 0.001) or water (mock) and samples were collected at 24 hpi. Leaves of transgenic plants carrying DEX-inducible AtMKK4DD in a sid2 background were infiltrated with 2 µM DEX or 0.1% ethanol and samples were collected at 24 hpi. The expression levels of PR1 (At2g14610) (A), Chitinase (At1g02360) (B) and CHS (At5g13930) (C), which represent Clusters I, II, and III, respectively, were determined by qRT-PCR. Bars represent means and standard errors of three biological replicates calculated using a mixed linear model. The vertical axis is the log2 expression level relative to Actin2 (At2g18780). Asterisks indicate significant differences from mock (P<0.01, two-tailed t-tests). (TIF) [file pgen.1004015.s010.tif]

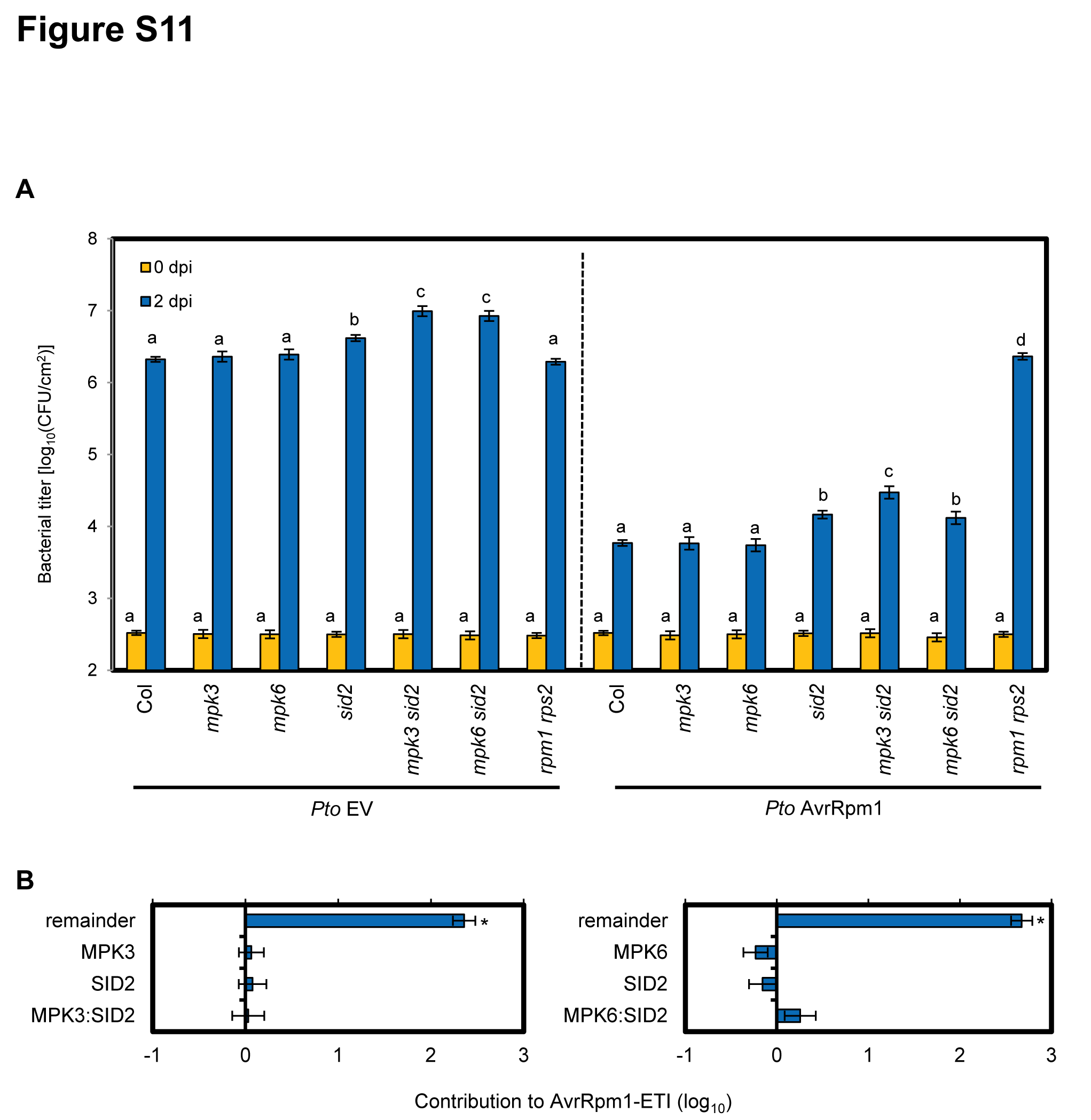

Supplement: Figure S11 — AvrRpm1-ETI. (A) The bacterial counts of Pto EV (left panel) or AvrRpm1 (right panel) (inoculation dose, OD600 = 0.0001) at 0 or 2 dpi in leaves of the indicated genotypes were measured. Bars represent means and standard errors of three independent experiments with at least 4 or 12 biological replicates for 0 dpi or 2 dpi, respectively. Statistically significant differences are indicated by different letters per strain per dpi (P<0.01, two-tailed t-tests). (B) The signaling allocations for AvrRpm1-ETI shown in (A, 2 dpi) were estimated for MPK3 and SID2 (left panel) or MPK6 and SID2 (right panel). Bars represent means and standard errors determined using a mixed linear model. Asterisks indicate significant effects or interaction (P<0.01). (TIF) [file pgen.1004015.s011.tif]
