# Supplementary material for: Dual Regulation of Gene Expression Mediated by Extended MAPK Activation and Salicylic Acid Contributes to Robust Innate Immunity in Arabidopsis thaliana
Source: PLoS Genet. 2013 Dec 12;9(12):e1004015. doi: 10.1371/journal.pgen.1004015 (PMC3861249; doi:10.1371/journal.pgen.1004015)
Supplement: Table S4 — Primers used in this study. (DOCX) [file pgen.1004015.s015.docx]

**Supplemental tables**

**Table S4. Primers used in this study**

Genotyping

|  | Primer (5' to 3') | |  |
| --- | --- | --- | --- |
| mutation | Forward | Reverse | PCR product size |
| *mpk3* | GAGCCATCACTGTCTGCTTCGTA | GCAGTTGAACAAGCTCTGAATCAC | 748 bp (WT) |
| *mpk3* | GGAACAACACTCAACCCTATCTCG | GCAGTTGAACAAGCTCTGAATCAC | 472 bp (*mpk3*) |
| *mpk6-2* | CTCGGTCTGTTTCTTTAGTGCTTC | GTTGGCGTTTGCAACGTGTAAAACAT | 690 bp (WT) |
| *mpk6-2* | GGAACAACACTCAACCCTATCTCG | GTTGGCGTTTGCAACGTGTAAAACAT | 680 bp (*mpk6-2*) |
| *npr1-1* | ATGTCTCGAATGTACATAAGGCACTTG | ATGAGTGCGGTTCTACCTTCCAAA | 304 bp |
| *sid2-2* | TTCTTCATGCAGGGGAGGAG | AAGCAAAATGTTTGAGTCAGCA | 879 bp (WT) 581 bp (*sid2-2*) |
|  | CAACCACCTGGTGCACCAGC |  |  |

| mutation | Restriction Enzyme | fragments in WT (bp) | fragments in mutant (bp) |
| --- | --- | --- | --- |
| *npr1-1* | *NlaIII* | 97 and 207 bp | 304 bp |

qRT-PCR

|  | Primer (5' to 3') | |
| --- | --- | --- |
| *PR1* | CGGAGCTACGCAGAACAACT | CTCGCTAACCCACATGTTCA |
| *FRK1* | GGAAGCGGTCAGATTTCAAC | AGCTTGCAATAGCAGGTTGG |
| *Actin2* | AGTGTCTGGATCGGTGGTTC | CCCCAGCTTTTTAAGCCTTT |
| *CHS* | AAGTCGACAATTCGGAAACG | ACTTCGACCACCACGATGTC |
| *Chtinase* | TGGCTCAGATTTCACACGAG | TTAGGGAAACAAGGCCACTG |
| *MKK4* | GGCGTCTTCAACGAACACTA | GAGAACTCGGACGGTGAATC |
| *MKK5* | CGCCGCTAAAAGCTTATCC | CGTCTCACGGTATCTTCGTG |
